# A Versatile Strategy for Genetic Manipulation of Cajal–Retzius Cells in the Adult Mouse Hippocampus

**DOI:** 10.1523/ENEURO.0054-23.2023

**Published:** 2023-10-16

**Authors:** Rebekah van Bruggen, Zain H. Patel, Mi Wang, Terry R. Suk, Maxime W. C. Rousseaux, Qiumin Tan

**Affiliations:** 1 Department of Cell Biology, University of Alberta, Edmonton, Alberta T6G 2H7, Canada; 2 Brain and Mind Research Institute, University of Ottawa, Ottawa, Ontario K1H 8M5, Canada; 3 Department of Cellular and Molecular Medicine, University of Ottawa, Ottawa, Ontario K1H 8M5, Canada; 4 Eric Poulin Center for Neuromuscular Diseases, University of Ottawa, Ottawa, Ontario K1H 8M5, Canada; 5 Ottawa Institute of Systems Biology, University of Ottawa, Ottawa, Ontario K1H 8M5, Canada; 6 Women and Children’s Health Research Institute, University of Alberta, Edmonton, Alberta T6G 1C9, Canada

**Keywords:** adeno-associated virus, adult hippocampus, Cajal–Retzius cells, Δ*Np73-Cre*, genetic modification, neonatal intracerebroventricular injection

## Abstract

Cajal–Retzius (CR) cells are transient neurons with long-lasting effects on the architecture and circuitry of the neocortex and hippocampus. Contrary to the prevailing assumption that CR cells completely disappear in rodents shortly after birth, a substantial portion of these cells persist in the hippocampus throughout adulthood. The role of these surviving CR cells in the adult hippocampus is largely unknown, partly because of the paucity of suitable tools to dissect their functions in the adult versus the embryonic brain. Here, we show that genetic crosses of the Δ*Np73-Cre* mouse line, widely used to target CR cells, to reporter mice induce reporter expression not only in CR cells, but also progressively in postnatal dentate gyrus granule neurons. Such a lack of specificity may confound studies of CR cell function in the adult hippocampus. To overcome this, we devise a method that not only leverages the temporary CR cell-targeting specificity of the Δ*Np73-Cre* mice before the first postnatal week, but also capitalizes on the simplicity and effectiveness of freehand neonatal intracerebroventricular injection of adeno-associated virus. We achieve robust Cre-mediated recombination that remains largely restricted to hippocampal CR cells from early postnatal age to adulthood. We further demonstrate the utility of this method to manipulate neuronal activity of CR cells in the adult hippocampus. This versatile and scalable strategy will facilitate experiments of CR cell-specific gene knockdown and/or overexpression, lineage tracing, and neural activity modulation in the postnatal and adult brain.

## Significance Statement

High-throughput and specific tools for genetic manipulation of neuronal subtypes *in vivo* are desirable for scalable experiments and accurate data interpretation. However, limitations in available tools present a challenge for certain cell types, such as Cajal–Retzius cells, a class of transient neurons of which a portion persists in the adult brain. Highlighting the limitation of Cre-driver mouse lines because of loss of specificity in adulthood, we demonstrate the use of neonatal intracerebroventricular delivery of adeno-associated viral vectors to specifically manipulate Cajal–Retzius cells in the adult hippocampus. Our strategy offers a framework to address similar issues with experiment throughput and specificity of other neuronal subtypes.

## Introduction

Cajal–Retzius (CR) cells are a group of early-born glutamatergic neurons that populate the embryonic cortex (Squarzoni et al., 2015; Causeret et al., 2021; Vílchez-Acosta et al., 2022). Originating from four distinct progenitor domains of the brain, including the ventral pallium, the septum, the thalamic eminence, and the cortical hem, CR cells first appear at approximately embryonic day 9.5 (E9.5) in mice. They then migrate tangentially to cover the entire surface of the telencephalon. Their final destination is determined by their ontogenic origin and migration path, such that a particular brain region is predominantly occupied by CR cells from the same progenitor niche. A prominent example is the mouse hippocampus, which is populated almost elusively by cortical hem-derived CR cells (Yoshida et al., 2006; Louvi et al., 2007). Once in residence, CR cells control cortical neuron migration via secretion of the glycoprotein reelin (Meyer et al., 2004; Yoshida et al., 2006; Tissir et al., 2009; Amelio et al., 2020; Vílchez-Acosta et al., 2022). They also regulate cortical and hippocampal circuits through their connectivity in local networks (Anstötz et al., 2022; Genescu et al., 2022; Riva et al., 2023).

CR cells can be readily identified by a few selective markers including reelin (RELN) and p73 (TRP73), of which the latter is considered to be the most specific (Anstötz and Maccaferri, 2020). Morphologically distinct from other neurons by virtue of their smaller cell body and characteristic “tadpole” shape (Anstötz et al., 2016; Anstötz and Maccaferri, 2020), CR cells can also be recognized based on their distinct locations within the brain, particularly in the marginal zone of the embryonic neocortex (Del Río et al., 1996; Gil et al., 2014; Ma et al., 2014; Elorriaga et al., 2023), as well as along the hippocampal fissure and the molecular layer of the dentate gyrus (DG; Anstötz et al., 2016; Causeret et al., 2021; Glærum et al., 2022). Beyond protein markers and morphologic properties, an array of transgenic reporter or Cre-expressing mouse lines have been used for the study of CR cells in the developing brain (for review, see Causeret et al., 2021). For example, the Δ*Np73-Cre* mouse strain drives Cre expression in CR cells derived from the cortical hem, septum, and the thalamic eminence (Tissir et al., 2009), while the *Wnt3a-Cre* mouse line is restricted to cortical hem-derived CR cells (Yoshida et al., 2006). Though powerful, these genetic tools also nonspecifically label or target other cells (for review, see Causeret et al., 2021), which may cloud data interpretation.

CR cells have long been known as transient neurons as a majority of them undergo programmed cell death during development. In humans, massive CR cell death occurs around gestational weeks 23–28 (Meyer and González-Gómez, 2018a, b), with some cells persisting in layer 1 of the neocortex (Marín-Padilla, 1990; Martínez-Cerdeño et al., 2002; Martínez-Cerdeño and Clascá, 2002; Martínez-Cerdeño and Noctor, 2014) and the hippocampus (Blümcke et al., 1996, 1999). In mice, very few (∼5%) CR cells persist in the adult neocortex. However, a larger portion (20–30%) of CR cells remain in the hippocampus throughout adulthood (Anstötz et al., 2016, 2018a). The persistence of CR cells in the adult hippocampus is often overlooked, which may be partly because of the presumption of their complete disappearance. Because of this assumption, most of the available genetic tools for the study of CR cells have not been thoroughly characterized regarding their cell-type specificity in the adult brain, leading to a paucity of suitable tools to specifically manipulate persistent CR cells. Moreover, as CR cells have critical roles in embryonic brain development (Causeret et al., 2021; Elorriaga et al., 2023), methods that confer specific labeling or targeting of postnatal CR cells without affecting their embryonic counterparts are especially desirable for the dissection of their contributions in the adult brain. Here, we describe a simple and versatile strategy to genetically manipulate postnatal and adult CR cells by introducing Cre-dependent adeno-associated virus (AAV) constructs to neonatal Δ*Np73-Cre* mice via freehand intracerebroventricular injections. This method is relatively high throughput and low cost since it does not require special surgery and injection equipment. Our strategy will facilitate experiments that enhance our understanding of the function of CR cells in the early postnatal and adult hippocampus.

## Materials and Methods

### Mice

Δ*Np73-Cre* hemizygous mice (Tissir et al., 2009) and *Wnt3a-Cre* hemizygous mice (Yoshida et al., 2006) were a gift from Alessandra Pierani (Université Paris Cité, Paris, France) and were kept as hemizygous; only hemizygous Cre mice were used throughout this study. *LSL-tdTomato* [B6.Cg-Gt(ROSA)26Sor^tm9(CAG-tdTomato)Hze^/J (Ai9); stock #007909; Madisen et al., 2010], *LSL-ArchT-EGFP* [B6.Cg-Gt(ROSA)/26Sor^tm40.1(CAG-aop3/EGFP)Hze^/J, stock #021188; Daigle et al., 2018], *LSL-2XChETA-P2A-tdTomato* [B6;129-Gt(ROSA)26Sor^tm1(CAG-COP4^*^E123T^*^H134R,-tdTomato)Gfng^/J, stock #017455; Ting and Feng, 2013], and *LSL-HA-hM3D* [B6N;129-Tg(CAG-CHRM3*,-mCitrine)1Ute/J, stock #026220; Zhu et al., 2016] mice were obtained from The Jackson Laboratory. Mice were group housed in a 12 h light/dark cycle, with all experiments performed during the light period. For genotyping animals between P7 and P17, tail biopsy samples were taken at the time the mice were killed, followed by tissue lysis and genotyping PCRs (Table 1). For genotyping animals older than P17, ear notch biopsy samples were taken between P14 and P18, and only animals with the desired genotypes were kept and weaned from the parents at P21. The genotypes of these mice were confirmed again by taking tail biopsy samples at the time mice were killed. Both male and female mice were used for experiments. Detailed information regarding the number and sex of animals used in each experiment is provided in Table 2. Ages are indicated where applicable. All animal procedures were performed in accordance with the animal care committee regulations of the University of Alberta and the University of Ottawa.

**Table 1:** List of genotyping PCR primers used in this study

Gene/allele target	Primers (5′−3′)	PCR product size
Cre (generic)	CCGGGCTGCCACGACCAA	490 bp
	GGCGCGGCAACACCATTTTT	
*LSL-tdTomato*	AAG GGA GCT GCA GTG GAG TA	Mutant = 196 bp
	CCG AAA ATC TGT GGG AAG TC	WT = 297 bp
	GGC ATT AAA GCA GCG TAT CC	
	CTG TTC CTG TAC GGC ATG G	
*LSL-HA-hM3D*	CGCCACCATGTACCCATAC	Transgene/mutant = 204 bp
	GTGGTACCGTCTGGAGAGGA	Internal positive control/WT = 297 bp
	AAGGGAGCTGCAGTGGAGTA	
	CCGAAAATCTGTGGGAAGTC	
*LSL-ArchT*	AAG GGA GCT GCA GTG GAG TA	Mutant = ∼300 bp
	CCG AAA ATC TGT GGG AAG TC	WT = 297 bp
	ATT GCA TCG CAT TGT CTG AG	
	CCG AAA ATC TGT GGG AAG TC	
*LSL-ChETA*	CACTTGCTCTCCCAAAGTCG	Mutant = ∼300 bp
	TACTCCGAGGCGGATCACAAGC	WT = 452 bp
	ACTAGTCAATAATCAATGTCGACCGG	
Δ*NP73-Cre* specific	ATTCTCCCACCGTCAGTACG	WT = 328 bp
	GAATGCCAACTCTCAGTCCG	KO = 796 bp
	GTCTCTCTGAACCCCAACCA	

### Adeno-associated viruses

*pAAV-CAG-YFP* was generated as described previously (Rousseaux et al., 2018). *pAAV-hSyn-DIO-hM3D(Gq)-mCherry* was a gift from Bryan Roth [the University of North Carolina School of Medicine, Viral Prep #44361-AAV8, Addgene (http://n2t.net/addgene:44361); RRID:Addgene_44 361). *pAAV-EF1α-double floxed-hChR2(H134R)-mCherry-WPRE-HGHpA* was a gift from Karl Deisseroth [Stanford University, Viral Prep #20297-AAV8, Addgene (http://n2t.net/addgene:20297); RRID:Addgene_20 297]. Viruses were aliquoted and stored at −80°C until use. Thawed viruses were kept at 4°C and used within a week. When necessary, viruses were diluted to the desired titers using PBS.

### Neonatal intracerebroventricular injections

Neonatal intracerebroventricular injections were performed as previously described with minor modifications (Kim et al., 2013). Within 6 h after birth, the newborn pups and dam were transported to the surgery suite in their home cage. Half of the litter was removed from the cage and transferred to a biological safety cabinet. One at a time, each pup was anesthetized using hypothermia by placing the pup on a wet paper towel on ice. When the pup no longer responded to tactile stimulation, the animal was placed on a cold flat surface and the cranial surface was disinfected with a 70% ethanol wipe (catalog #326910, Becton Dickinson Canada). Using a gas-tight syringe (catalog #361025642, Hamilton) with a 32 gauge 1.25 cm needle (catalog #7762–03, Hamilton), 2 μl of AAV was injected into the lateral ventricles of each hemisphere. The injection sites were cleaned with a 70% ethanol wipe to remove any surface contamination. The pup was then placed on a 37°C heating pad until cardiac output improved, as evidenced by a bright pink skin color, and the pup regained mobility and responded to tactile stimulation. The pup was then kept warm in bedding on the heating pad until the remaining half of the litter was completed. The injected pups were then returned to the home cage together, at the same time removing the second half of the litter, thereby endeavoring to reduce stress on the dam. The remaining pups were injected with the virus as aforementioned. Once all pups were injected with the virus, the cages were then promptly returned to the housing suite. Pups were monitored daily for 1 week to ensure the absence of complications.

### Clozapine-*N*-oxide injection

At 7 weeks of age, AAV8/*hSyn-DIO-hM3D-mCherry* injected mice were intraperitoneally injected with 5 mg/kg clozapine-*N*-oxide (CNO; 4 mg/ml stock solution; catalog #6329/10, Tocris Bioscience) or sterile saline (0.9% NaCl; catalog #JB1324, Baxter). Two hours after the intraperitoneal injection, the brain tissues were collected as described below.

### Tissue preparations

Animals were deeply anesthetized via injection of sodium pentobarbital (240 mg/ml, i.p.; Euthanyl, Bimeda-MTC), then transcardially perfused with PBS (catalog #BP399-20, Fisher Bioreagents) followed by 4% paraformaldehyde in PBS (catalog #19202, Electron Microscope Sciences). The brains were removed and postfixed in 4% paraformaldehyde overnight at 4°C, followed by sequential submersion in 15% and then 30% sucrose for 24 h at 4°C for each change. Brain tissue was cut coronally using a brain matrix, followed by cryoembedding into Optimal Cutting Temperature compound (catalog #4585, Fisher HealthCare) and subsequently frozen at −80°C. Coronal brain sections (40 μm thick) were cut using a cryostat (catalog #CM1520, Leica Microsystems) and kept at 4°C in PBS with 0.02% sodium azide (catalog #7144.8–16, BICCA) as a preservative. Coronal sections were then transferred onto Superfrost Plus microscope slides (12–550-5, Thermo Fisher Scientific) and air dried overnight. Once the slides were dry, they were used for immunofluorescence staining or stored at −80°C.

### Nissl staining and immunofluorescence studies

Nissl (cresyl violet) staining was performed using standard protocols. For immunofluorescence studies, slides were postfixed in 10% phosphate buffered formalin (catalog #SF100-4, Fisher Chemicals) for 10 min at room temperature and then washed in PBS. Antigen retrieval was performed with a citric acid-based antigen unmasking solution (catalog #H-3300, Vector Laboratories) for 30 min in a 95°C water bath. Once the slides were cooled to room temperature, they were washed twice with PBS, permeabilized with PBST (PBS + 0.3% Triton X-100; catalog #BP151-500, Fisher Bioreagents) for 20 min at room temperature and then blocked with 5% normal donkey serum (catalog #D9663-10ML, Sigma-Aldrich) diluted in PBST (blocking solution) for 20 min at room temperature. Primary antibodies were diluted in blocking solution, added onto the slides, and incubated overnight at 4°C in a humid chamber. Sections were washed three times with PBST before incubating for 2 h at room temperature in secondary antibody diluted in blocking buffer. Afterward, the slides were washed in PBST then PBS, and autofluorescence was quenched using Vector TrueVIEW autofluorescence quenching kit (catalog #SP8400, Vector Laboratories), prepared as per manufacturer instructions for 2 min at room temperature. The slides were washed, counterstained with DAPI (5 μg/ml; catalog #D3571, Thermo Fisher Scientific) for 10 min of incubation at room temperature, then washed with PBS. The slides were then mounted using VECTASHIELD Vibrance Antifade Mounting Medium (catalog #H170010, Vector Laboratories) and covered with a coverslip. The slides were left to dry overnight, sealed with transparent nail polish, then further dried before being imaged with a confocal microscope.

### Antibodies

The following primary antibodies were used for immunofluorescence staining: goat anti-tdTomato (1:500; catalog #AB8181-200, SICGEN; RRID:AB_2722750); rabbit anti-TRP73 (1:500; catalog #ab40658, Abcam; RRID:AB_776999); mouse anti-RELN (1:500; catalog #MAB5364, MilliporeSigma; RRID:AB_1293544); mouse anti-RELN (1:500; catalog #ab78540, Abcam; RRID:AB_1603148); rabbit anti-CALB1 (1:500; catalog #CB38, Swant; RRID:AB_10000340); mouse anti-DCX (1:25; catalog #sc-271390, Santa Cruz Biotechnology; RRID:AB_10610966); and goat anti-EGFP/YFP (yellow fluorescent protein; 1:500; catalog #AB0020-500, SICGEN; RRID:AB_2333100). The secondary antibodies used were as follows: donkey anti-goat Alexa Fluor 555 (1:1000; catalog #A21432, Thermo Fisher Scientific; RRID:AB_2535853); donkey anti-rabbit Alexa Fluor 488 (1:1000; catalog #A21206, Thermo Fisher Scientific; RRID:AB_2535792); and donkey anti-mouse Alexa Fluor 647 (1:1000; catalog #A31571, Thermo Fisher Scientific; RRID:AB_162542).

### Confocal microscopy

Immunofluorescent images were taken using a laser-scanning confocal microscope (model LSM 700, Zeiss). For adult (>5 weeks old) mouse brains, three coronal sections from each animal spanning the dorsal dentate gyrus at bregma −1.46, −1.94, and −2.46 mm were selected for imaging. For younger mice, comparable anatomic sections were chosen for imaging. Tiled and *z*-stacked images were acquired for each animal.

### Data analyses and statistical method

Cell counting and area measurements were performed using Fiji ImageJ software (version 1.53; Schindelin et al., 2012). The hippocampal fissure area was defined as 60 μm above and below the hippocampal fissure, as previously described (Pahle et al., 2020). CR cells positive for TRP73 expression were counted along the entire hippocampal fissure or the entire molecular layer ventral to the infrapyramidal blade of the dentate gyrus (lower molecular blade) from at least three sections per animal. Cell densities were normalized to the length of the hippocampal fissure and/or the length of the lower molecular layer. The variation index was calculated using the difference of cell densities between the two hemispheres divided by the sum of the densities. Animals without any tdTomato-expressing granule neurons (three of seven mice at 7 weeks of age) were excluded from the granule neuron variation index calculation as they resulted in an invalid formula for the variation index calculation. Statistical analyses were performed using GraphPad Prism [version 9.4.1; GraphPad Software (www.graphpad.com)]. Detailed statistics for all analyses in the article are presented in Table 2.

**Table 2:** Summary of statistical analysis

Experiment	Animal number and sex	Test	*p*-value	*t*, df	*F*, dfn, dfd
Figure 2*B*	P7: *N* = 6 (3 females and 3 males); P17: N= 4 (2 females and 2 males); 4W: *N* = 4 (2 females and 2 males); 7W: *N* = 7 (3 females and 4 males)	* *			
tdT^+^ CR cell density, hippocampal fissure		Nested one-way ANOVA	<0.0001		79.85, 3, 59
Figure 2*B*					
tdT^+^ CR cell density, lower molecular layer		Nested one-way ANOVA	**0.0095; ****<0.0001		178.3, 3, 59
Figure 2*B*					
tdT^+^ granule neurons, suprapyramidal blade		Nested one-way ANOVA	*0.0367; ***0.0003		6.819, 3, 59
Figure 2*B*					
tdT^+^ granule neurons, infrapyramidal blade		Nested one-way ANOVA	**0.0024		5.054, 3, 59
Figure 2*B*			P7 vs P17: *0.0491; P7 vs 4W: ****<0.0001		
CR cell-targeting specificity		Nested one-way ANOVA	P7 vs 7W: *0.0369; 4W vs 7W: *0.0179		9.611, 3, 59
Extended Data Figure 2-1					
tdT^+^ CR cell density, neocortex		Nested one-way ANOVA	<0.0001		148.7, 3, 59
Extended Data Figure 2-3					
tdT^+^ granule neurons, suprapyramidal blade, P17		Nested *t* test	0.1918	*t* = 1.400, df = 10	1.960, 1, 10
Extended Data Figure 2-3					
tdT^+^ granule neurons, suprapyramidal blade, 4W		Nested *t* test	0.2669	*t* = 1.176, df = 10	1.382, 1, 10
Extended Data Figure 2-3					
tdT^+^ granule neurons, suprapyramidal blade, 7W		Nested *t* test	0.4237	*t* = 0.8177, df = 19	0.6686, 1, 19
Extended Data Figure 2-3					
tdT^+^ granule neurons, infrapyramidal blade, P17		Nested *t* test	0.212	*t* = 1.333, df = 10	1.777, 1, 10
Extended Data Figure 2-3					
tdT^+^ granule neurons, infrapyramidal blade, 4W		Nested *t* test	0.5247	*t* = 0.6592, df = 10	0.4345, 1, 10
Extended Data Figure 2-3					
tdT^+^ granule neurons, infrapyramidal blade, 7W		Nested *t* test	0.4111	*t* = 0.8405, df = 19	0.7064, 1, 19
Figure 3*B*	4W: *N* = 4 (2 females and 2 males); 7W: *N* = 7 (2 females and 2 males)				
tdT^+^ cells, 4W		Welch's *t* test	0.1813		*t* = 1.730, df = 3.025
Figure 3*B*					
tdT^+^ cells, 7W		Welch's *t* test	0.0424		*t* = 3.362, df = 3.060
Figure 4*C*	P14: *n* = 1 female and 1 male; 5W: *n* = 1 female and 3 males	** **	*** ***	** **	** **
CR cell density		Nested *t* test	<0.0001	*t* = 11.02, df = 19	121.4, 1, 19
Figure 4*C*					
Granule neuron density		Nested *t* test	0.2771	*t* = 1.219, df = 5	1.487, 1, 5
Figure 4*D*					
CR cell density (hippocampal fissure)		Nested one-way ANOVA	*0.0194; **0.0013		14.25, 2, 9
Figure 4*D*					
CR cell density (lower molecular layer)	*ArchT^ΔNp73-Cre^*: *n* = 3 males	Nested one-way ANOVA	**0.0015; ***0.0003		23.03, 2, 9
Figure 4*D*	*ChETA^ΔNp73-Cre^*: *n* = 2 females and 1 male				
Granule neuron density (suprapyramidal blade)	*hM3Dq^ΔNp73-Cre^*: *n* = 1 female and 3 males	Nested one-way ANOVA	**0.0017; ****<0.0001		14.34, 2, 36
Figure 4*D*					
Granule neuron density (infrapyramidal blade)		Nested one-way ANOVA	***0.0003; ****<0.0001		21.16, 2, 36
Figure 4*D*					
CR cell targeting specificity		Nested one-way ANOVA	****<0.0001		28.05, 2, 36
Figure 4*E*					
Variation between hemispheres (*ArchT^ΔNp73-Cre^*)	*n* = 3 males	Nested *t* test	0.1259	*t* = 2.545, df = 2	6.478, 1, 2
Figure 4*E*					
Variation between hemispheres (*hM3Dq^ΔNp73-Cre^*)	*n* = 1 female and 3 males	Nested *t* test	0.1308	*t* = 2.486, df = 2	6.180, 1, 2
Extended Data Figure 4-2*D*					
CR cell density (Layer 1)	*ArchT^ΔNp73-Cre^*: *n* = 3 males	Nested one-way ANOVA	Not significant		1.863, 2, 17
	*ChETA^ΔNp73-Cre^*: *n* = 2 females and 1 male				
	*hM3Dq^ΔNp73-Cre^*: *n* = 1 female and 3 males				
Figure 5*B*	1.0 × 10^11^: *N* = 5 (1 female and 4 males); 1.0 × 10^12^: *N* = 4 (1 female and 3 males); 1.0 × 10^13^: *N* = 5 (4 females and 1 male);	** **	*** ***		** **
Percentage of mCherry^+^ CRs, hippocampal fissure		Nested one-way ANOVA	**0.0013; ****<0.0001		261.2, 2, 6
Figure 5*B*					
Percentage of mCherry^+^ CRs, lower molecular layer		Nested one-way ANOVA	**0.0016; ***0.0001; ****<0.0001		152.8, 2, 6
Figure 5*B*					
Percentage of mCherry^+^ granule neurons		Nested one-way ANOVA	10^11 vs. 10^13: 0.0032 (**); 10^12 vs. 10^13: 0.0025 (**)		8.613, 2, 39
Extended Data Figure 5-3*C*	1.0 × 10^11^ at P14: *N* = 5 (2 females and 3 males); 1.0 × 10^12^ at P14: *N* = 6 (1 female and 5 males); 1.0 × 10^13^ at P14: *N* = 3 (2 females and 1 male); 1.0 × 10^12^ at 7W: *N* = 3 (3 females)				
Percentage of mCherry^+^ CRs, hippocampal fissure		Nested one-way ANOVA	all not significant		0.8421, 3, 47
Extended Data Figure 5-3*C*					
Percentage of mCherry^+^ CRs, lower molecular layer		Nested one-way ANOVA	all not significant		0.5963, 3, 47
Extended Data Figure 5-3*C*					
Number of mCherry^+^ granule neurons		Nested one-way ANOVA	all not significant		1.443, 3, 8
Extended Data Figure 5-3*D*	WT: *N* = 3 (2 females and 1 male)				
Number of mCherry^+^ granule neurons	Δ*Np73-Cre*: *N* = 5 (4 females and 1 male)	Nested *t* test	0.0313		5.290, 1, 22
Figure 6*B*	WT: *n* = 3 (3 females)	** **	*** ***	*** ***	** **
Percentage of mCherry^+^ CRs, hippocampus	Δ*Np73-Cre*: *n* = 10 (3 females and 7 males)	Nested *t* test	<0.0001	*t* = 11.61, df = 36	134.9, 1, 36
Figure 6*C*	P0 to >P14: *n* = 4 (1 female and 3 males)				
Percentage of mCherry^+^ granule neurons	P0 to >7W: *n* = 10 (3 females and 7 males)	Nested *t* test	0.0506	*t* = 2.017, df = 39	4.069, 1, 39
Figure 6*D*	*tdT*Δ*Np73-Cre*: *n* = 7 (3 females and 4 males)				
Reporter-expressing granule neurons at 7W	AAV-ChR2: *n* = 10 (3 females and 7 males)	Nested one-way ANOVA	***0.0006 and 0.0007		9.297, 2, 83
Figure 6*D*	AAV-hM3D: *n* = 12 (5 females and 7 males)				
CR targeting specificity		Nested one-way ANOVA	***0.0003 and 0.0009		9.563, 2, 83
Figure 6*E*	WT, P14: *n* = 6 (1 female and 5 males)				
Percentage of mCherry^+^ CRs, hippocampal fissure	WT, 7W: *n* = 3 (3 females)	Two-way ANOVA	Interaction: 0.2082		1.673, 1, 24
Figure 6*E*	Δ*Np73-Cre*, P14: *n* = 4 (1 female and 3 males)				
Percentage of mCherry^+^ CRs, lower molecular layer	Δ*Np73-Cre*, 7W: *n* = 10 (3 females and 7 males)	Two-way ANOVA	Interaction: 0.9824		0.0004962, 1, 24
Figure 7*B*		** **	*** ***	*** ***	** **
mCherry^+^ CRs at P14, hippocampal fissure	AAV/ChR2: *n* = 4 (1 female and 3 males)	Nested *t* test	<0.0001	*t* = 5.563, df = 19	30.94, 1, 19
Figure 7*B*	AAV/hM3D: *n* = 6 (1 female and 5 males)				
mCherry^+^ CRs at P14, lower molecular layer		Nested *t* test	0.0002	*t* = 4.696, df = 19	22.05, 1, 19
Figure 7*D*		** **			
mCherry^+^ CRs at 7W, hippocampal fissure	AAV/ChR2: *n* = 10 (3 females and 7 males)	Nested *t* test	0.0011	*t* = 3.422, df = 63	11.71, 1, 63
Figure 7*D*	AAV/hM3D: *n* = 12 (5 females and 7 males)				
mCherry^+^ CRs at 7W, lower molecular layer		Nested *t* test	0.0072	*t* = 2.779, df = 63	7.724, 1, 63
Figure 7*E*	P0 to >P14: *n* = 6 (1 female and 5 males)				
mCherry^+^ CRs at 7W, lower molecular layer	P0 to >7W: *n* = 12 (5 females and 7 males)	Nested *t* test	0.0011	*t* = 3.467, df = 52	12.02, 1, 52
Figure 7*F*	Wild type, P14: *n* = 8 (6 females and 2 males)				
Total CRs, hippocampal fissure	Wild type, 7W: *n* = 7 (2 females and 5 males)	Two-way ANOVA	Interaction: 0.4793		0.5136, 1, 29
Figure 7F	Δ*Np73-Cre*, P14: *n* = 6 (1 female and 5 males)				
Total CRs, lower molecular layer	Δ*Np73-Cre*, 7W: *n* = 12 (5 females and 7 males)	Two-way ANOVA	Interaction: 0.8317		0.04601, 1, 29
Extended Data Figure 7-1*C*					
Percentage of mCherry^+^ CRs, hippocampal fissure		Nested *t* test	0.2784	*t* = 1.098, df = 42	1.206, 1, 42
Extended Data Figure 7-1*C*	P0 to >P14: *n* = 8 (6 females and 2 males)				
Percentage of mCherry^+^ CRs, lower molecular layer	P0 to >7W: *n* = 7 (2 females and 5 males)	No test was performed as there was no variation among replicates			
Extended Data Figure 7-1*C*					
mCherry^+^ granule neuron density		No test was performed as there was no variation among replicates			
Extended Data Figure 7-2	Female, AAV/ChR2: *n* = 3				
Percentage of mCherry^+^ CRs at 7W, hippocampal fissure	Male, AAV/ChR2: *n* = 7	Nested one-way ANOVA	**0.0066		4.502, 3, 58
Extended Data Figure 7-2	Female, AAV/hM3D: *n* = 5				
Percentage of mCherry^+^ CRs at 7W, lower molecular layer	Male, AAV/hM3D: *n* = 7	Nested one-way ANOVA	Not significant		2.638, 3, 61
Figure 8*C*		** **	*** ***	*** ***	** **
Percentage of mCherry^+^ CR cells, hippocampal fissure		Nested *t* test	<0.0001	*t* = 10.30, df = 34	106.0, 1, 34
Figure 8*C*					
Percentage of mCherry^+^ CR cells, lower molecular layer	Saline: *n* = 5 (2 females and 3 males)	Nested *t* test	<0.0001	*t* = 10.55, df = 34	111.2, 1, 34
Figure 8*C*	CNO: *n* = 7 (3 females and 4 males)	** **			
Percentage of total^+^ CR cells, hippocampal fissure		Nested one-way ANOVA	****<0.0001		98.35, 3, 68
Figure 8*C*					
Percentage of total CR cells, lower molecular layer		Nested one-way ANOVA	****<0.0001		114.2, 3, 68
Extended Data Figure 8-1					
Percentage of total^+^ CR cells, hippocampal fissure	Saline: *n* = 5 (5 males)	Nested one-way ANOVA	Not significant		0.7748, 3, 8
Extended Data Figure 8-1	CNO: *n* = 2 (2 females)				
Percentage of total^+^ CR cells, lower molecular layer		Nested one-way ANOVA	Not significant		0.6000, 3, 36

W, Weeks; P, postnatal days; CR, Cajal-Retzius cells; WT, wild type; tdT, tdTomato; df, degree of freedom. dfn, degrees of freedom in the numerator; dfd, degrees of freedom in the denominator.

## Results

### Neither *Wnt3a-Cre* nor Δ*Np73-Cre* lines are specific to Cajal–Retzius cells in the hippocampus by the third postnatal week

**Figure 1.**
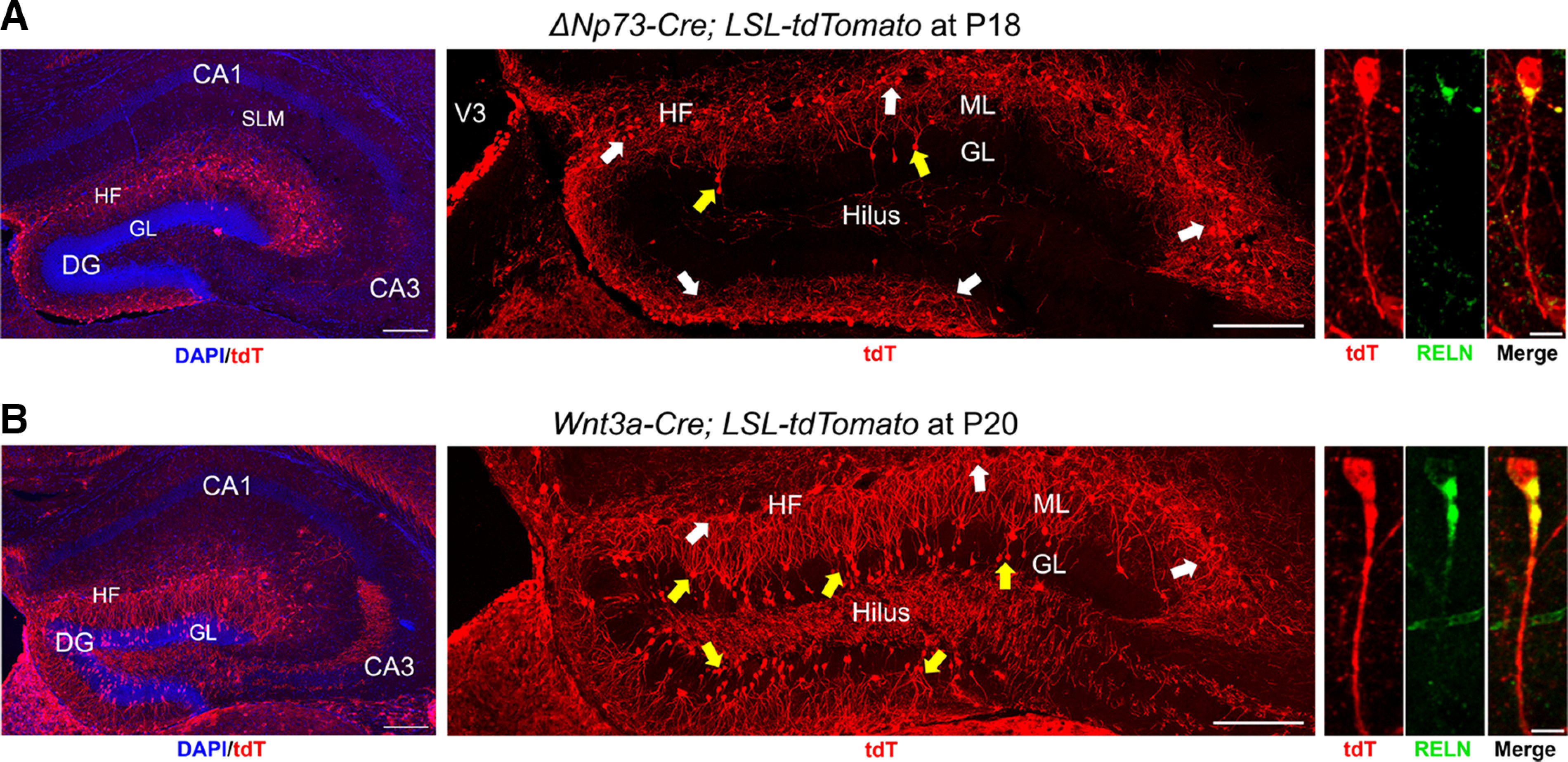
Characterization of tdTomato (tdT) reporter expression driven by the Δ*Np73-Cre* or the *Wnt3a-Cre* mouse line. ***A***, ***B***, Comparison of tdT reporter expression in the Δ*Np73-Cre; LSL-tdTomato* mice at P18 (***A***) and the *Wnt3a-Cre; LSL-tdTomato* mice at P20 (***B***). Left, Representative images show tdTomato expression in the hippocampus. In the hippocampus of both mouse lines, reporter expression is limited to the DG and along the hippocampal fissure (HF). Reporter expression was not detected in cornu ammonis (CA) regions. Scale bars, 200 μm. Middle, Higher-magnification images show reporter expression in the DG. In both transgenic lines, reporter expression is readily detectable in Cajal–Retzius cells (white arrows) located along the HF and in the dentate gyrus molecular layer (ML). While cells in the granular layer (GL; yellow arrows) of the Δ*Np73-Cre; LSL-tdTomato* mice are only occasionally observed to express tdTomato, there is extensive tdTomato expression in the granular layer in the *Wnt3a-Cre; LSL-tdTomato* mice. Scale bars, 200 μm. Right panels, Cajal–Retzius cells are identified by their unique tadpole-like morphology and the expression of RELN. Scale bars, 10 μm. SLM, Stratum lacunosum-molecular; V3, third ventricle.

Several Cre-driver transgenic mouse lines have been developed to study CR cells in the developing brain (Causeret et al., 2021). However, few studies have pursued the role of CR cells within the adult brain. To achieve this, we first set out to identify a transgenic mouse line that will allow us to specifically manipulate CR cells in the hippocampus. We assessed two popular mouse lines used to drive Cre expression in cortical hem-derived CR cells, the Δ*Np73-Cre* line (Tissir et al., 2009) and the *Wnt3a-Cre* line (Yoshida et al., 2006), by crossing them to the Cre-dependent *LSL-tdTomato* reporter mice. At P18, the Δ*Np73-Cre* line demonstrated high specificity in the hippocampus with tdTomato reporter expression mostly restricted to CR cells, which were characterized by their unique tadpole-like morphology, reelin expression, and localization along the hippocampal fissure and in the molecular layer of the dentate gyrus (Fig. 1*A*). In contrast, the *Wnt3a-Cre* line drove broad tdTomato expression outside of the CR cell domain, in what were likely DG granule neurons (Fig. 1*B*), consistent with previous reports (Quattrocolo and Maccaferri, 2014; Anstötz et al., 2018a) and the expression of *Wnt3* in the postnatal hippocampal neurogenic niche (Lie et al., 2005). As such, we opted to use the Δ*Np73-Cre* line for the remainder of this study.

**Figure 2.**
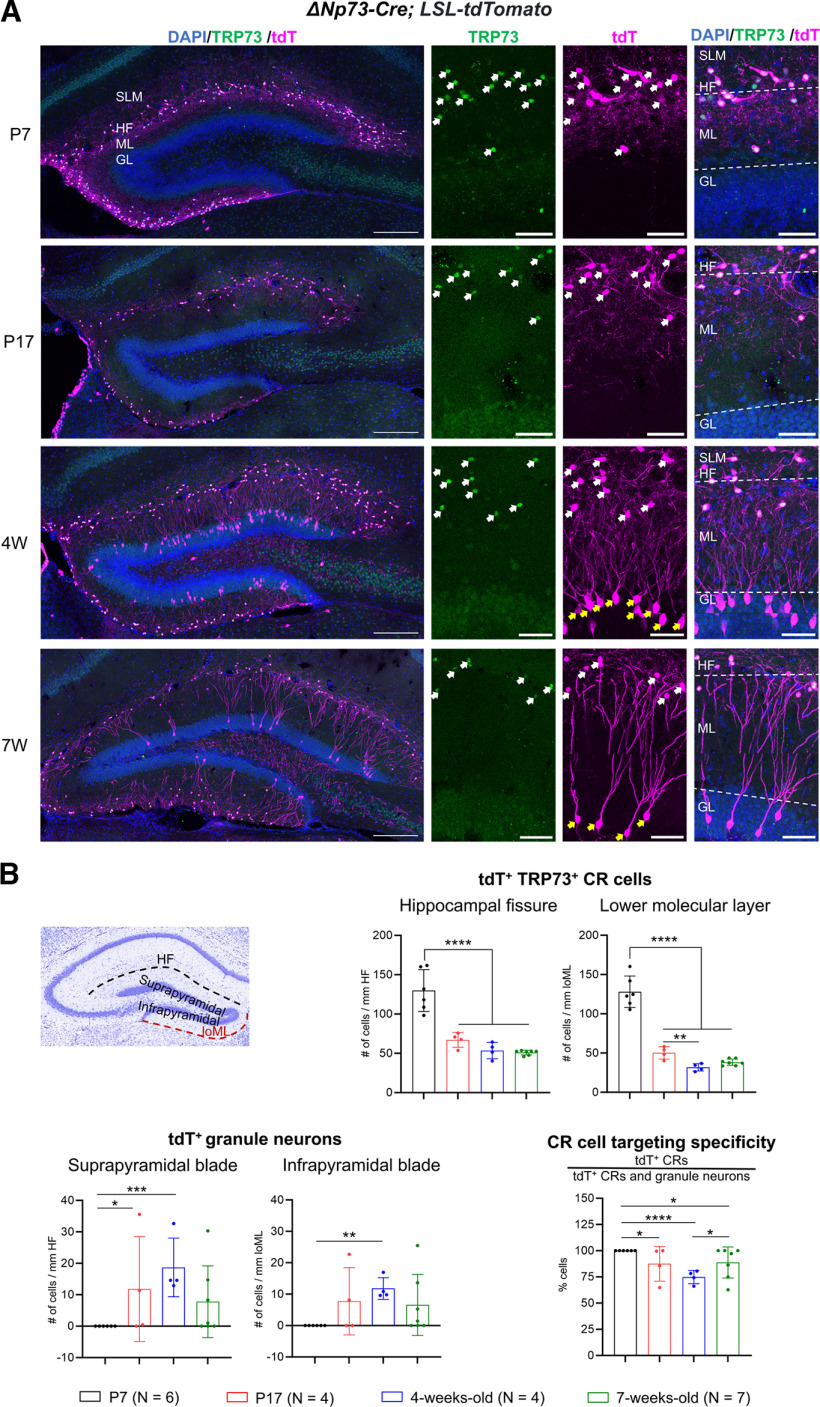
Reporter expression in the Δ*Np73-Cre; LSL-tdTomato* mice. ***A***, Left, Representative confocal images show tdTomato (tdT) reporter expression in the Δ*Np73-Cre; LSL-tdTomato* mice at P7, P17, 4 weeks (4W), and 7W. Right panels, High-magnification images are shown with coimmunostaining for tdTomato and the Cajal–Retzius (CR) cell marker TRP73. TRP73^+^ CR cells expressing tdTomato are marked with white arrows, while TRP73^–^ granule neurons of the dentate gyrus are marked with yellow arrows. SLM, Stratum lacunosum-molecular; HF, hippocampal fissure; GL, granular layer; ML, molecular layer. Scale bars: whole dentate gyrus, 200 μm; higher magnification, 20 μm. ***B.*** Left, An image of a Nissl-stained mouse brain coronal section depicts the areas of interest. loML, Lower ML (i.e., ML of the infrapyramidal blade). Right, Quantification of tdTomato^+^ CR cells and granule neurons, including quantification of CR cell-targeting specificity. Data are presented as scatter plots with all data points shown and error bars representing ±SD. Each data point is an individual animal, whereby three sections were measured for each animal. Statistical analyses were performed using nested one-way ANOVA with Tukey’s *post hoc* test. *p <* *0.05; **p <* *0.01; ***p <* *0.001; ****p <* *0.0001. The data that illustrate reporter expression in the neocortex are shown in Extended Data Figure 2-1. The images that demonstrate granule neuron identity are shown in Extended Data Figure 2-2. Analyses of the effect of sex on tdTomato-expressing granule neurons are provided in Extended Data Figure 2-3.

In the hippocampus of the Δ*Np73-Cre; LSL-tdTomato* mice, we occasionally observed non-CR cells labeled in the DG granular layer (Fig. 1*A*, yellow arrows). This prompted us to further characterize the extent of reporter expression in the hippocampus of these mice throughout postnatal development. At P7, tdTomato reporter expression was seen exclusively in CR cells in the DG (Fig. 2*A*). From P7 to P21, CR cells in the hippocampus undergo massive cell death, with ∼50% of the entire population being eliminated. CR cell number continues to decline until ∼5 weeks of age then stabilizes, leaving ∼20–30% of CR cells persisting throughout adulthood (Anstötz et al., 2018a). In agreement with this, we observed a drastic reduction of tdTomato-expressing CR cells along the hippocampal fissure and in the molecular layer from P7 to P17, which then stabilized by 4 weeks of age (Fig. 2*A*,*B*), suggesting that the Δ*Np73-Cre* and the *LSL-tdTomato* alleles do not overtly alter the time course of developmental programmed cell death of CR cells. In the neocortex, reporter expression was restricted to CR cells in layer 1, and CR cell density in 7-week-old mice was reduced to ∼5% of that in P7 mice (Extended Data Fig. 2-1).

10.1523/ENEURO.0054-23.2023.f2-1 Figure 2-1 Reporter expression is restricted to Cajal–Retzius neurons in the neocortex of the Δ*Np73-Cre; LSL-tdTomato* mice. Left, Representative confocal images show coimmunostaining of the tdTomato reporter and the CR cell marker TRP73 in P7, P17, and 4-week-old (4W) and 7W mice. Arrows point to CR cells. Scale bars, 100 μm. L, Layer. Right, Quantification of cortical layer 1 CR cell density at different ages demonstrates the developmental program cell death of CRs. Data are presented as a scatter plot with all data points shown. Each data point is an individual animal, whereby three sections were measured for each animal. Statistical analyses were performed using nested one-way ANOVA with Tukey’s *post hoc* test. ****p <* *0.0001. Download Figure 2-1, TIF file.

10.1523/ENEURO.0054-23.2023.f2-2 Figure 2-2 Recombination in mature granule neurons in the Δ*Np73-Cre; LSL-tdTomato* mice at 4 weeks of age. Coimmunostaining with the mature neuron marker CALB1 and the immature neuron marker DCX demonstrates that the tdTomato^+^ neurons in the granular layer (GL) are mature granule neurons (yellow arrowheads). Scale bars, 25 μm. ML, Molecular layer Download Figure 2-2, TIF file.

**Figure 3.**
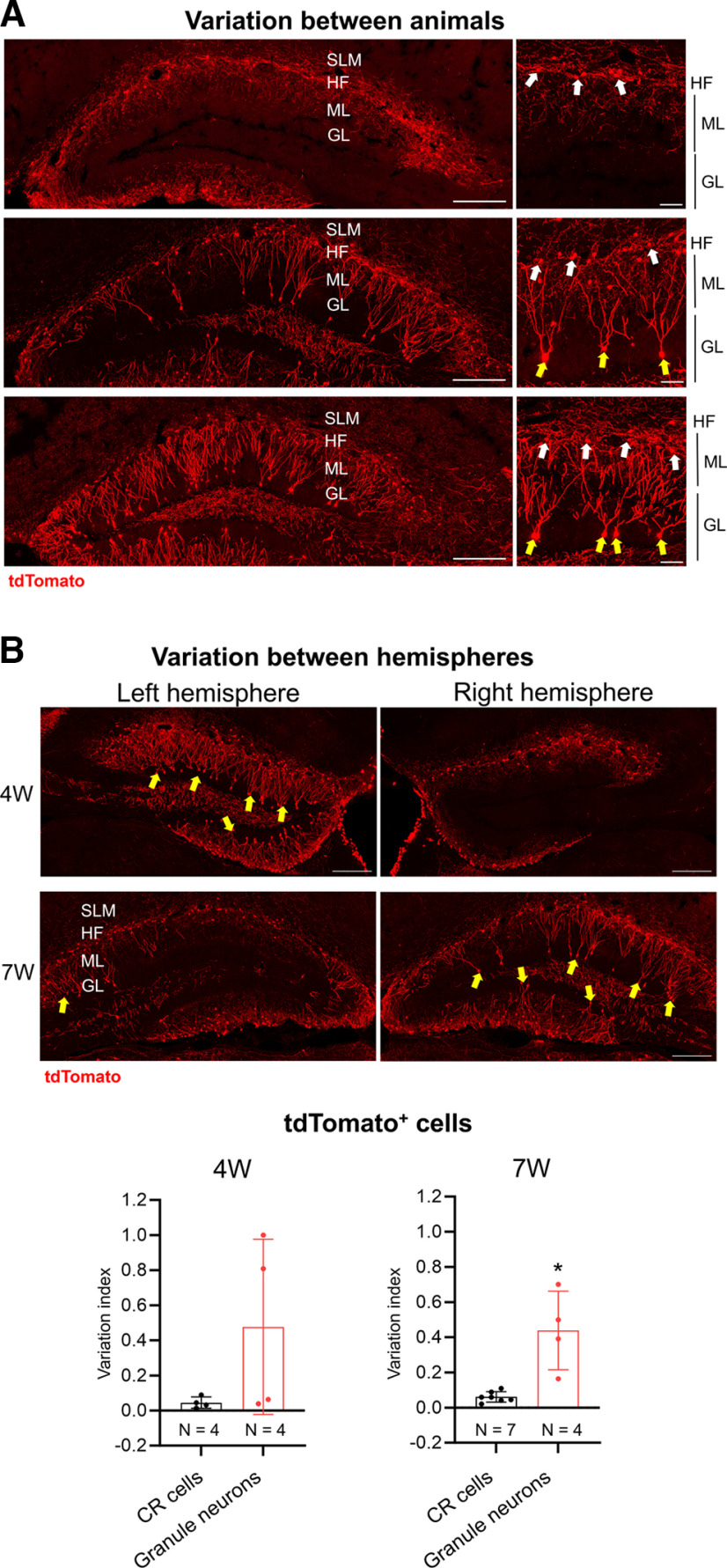
Variable expression of tdTomato reporter in 7-week-old Δ*Np73-Cre; LSL-tdTomato* mice. ***A***, Left, Representative confocal images show variable tdTomato expression in the DG between different animals at 7 weeks of age. Each image represents an individual animal. Scale bars, 200 μm. Right, Higher-magnification images show CR cells (white arrows) and dentate gyrus granule neurons (yellow arrows). Scale bars, 50 μm. ***B***. Representative confocal images show variable tdTomato expression between the two hemispheres of a 4-week-old and a 7-week-old animal. Yellow arrows point to granule neurons. Quantification of the variation indexes (the difference of cell densities between the two hemispheres divided by the sum of the cell densities) is shown at the bottom. Between the two hemispheres of 7-week-old animals, tdTomato expression in granule neurons is significantly more variable than that in CR cells. Data are presented as scatter plots with all data points shown and error bars representing ±SD. Each data point is an individual animal, whereby three sections were measured for each animal. Statistical analyses were performed using Welch’s *t* test. *p <* *0.05. SLM, Stratum lacunosum-moleculare; HF, hippocampal fissure; ML, molecular layer; GL, granular layer.

10.1523/ENEURO.0054-23.2023.f2-3 Figure 2-3 Analysis of potential sex effects on the variation of recombination in granule neurons in the Δ*Np73-Cre; LSL-tdTomato* mice. W, Weeks of age. Data are presented as scatter plots with all data points shown and error bars representing ±SD. Each data point is an individual animal, whereby three sections were measured for each animal. Statistical analyses were performed using nested *t* test. Download Figure 2-3, TIF file.

Coincidental with the postnatal pruning of the CR cell population, the DG undergoes protracted development. The DG develops from E13 to P15 in mice, with continuous neurogenesis throughout adulthood thereafter (Yu et al., 2014). At P7, when the DG is still developing, we did not find any cells in the granular layer with reporter expression (Fig. 2*A*,*B*). At P17, shortly after DG morphologic maturation is completed and when mature granule neurons begin to emerge (Hochgerner et al., 2018), reporter expression was detected in some non-CR cells in the DG granular layer. To determine the identity of these tdTomato-expressing non-CR cells, we immunostained for doublecortin (DCX) and calbindin (CALB1), which mark immature and mature DG granule neurons, respectively (Hourigan et al., 2021). We found them to be DCX^–^ but CALB1^+^, indicating these were mature granule neurons (Extended Data Fig. 2-2). There is a general trend that the numbers of these tdTomato-expressing non-CR cells increase after P17. By 4 weeks of age, we observed a significant increase in tdTomato-expressing granule neurons in the granular layer of the suprapyramidal and infrapyramidal blades of the dentate gyrus, as well as a concomitant decrease in CR cell-targeting specificity (Fig. 2*B*). Of note, reporter expression in granule neurons between different mice varied substantially (Figs. 2*B*, 3*A*), although these animals were housed under the same conditions and were handled and genotyped in a similar way. These animals were from different litters, and we did not detect any sex effects on the variability (Extended Data Fig. 2-3). We also noted significant variation between the DGs of both hemispheres within the same mouse (Fig. 3*B*). We further quantified this variation using the variation index, which is the difference of cell densities between the two hemispheres divided by the sum of the densities. If the two hemispheres have similar densities of cells, then the variation index would be near zero. Our analysis showed that there was limited between-hemisphere variation for CR cells in Δ*Np73-Cre; LSL-tdTomato* mice, whereas the variation indexes for granule neurons were significantly higher (Fig. 3*B*). This suggests that the Δ*Np73-Cre* allele may drive recombination in mature granule neurons because of individual differences (e.g., neurobehavior, physical activity, and other individual experiences), rather than because of transient Cre expression in a regulated developmental program. Overall, our genetic crosses indicate that, while both *Wnt3a-Cre* and Δ*Np73-Cre* lines induce robust reporter expression in CR cells, they also lead to additional recombination in postnatal DG granule neurons by the third postnatal week.

### The Δ*Np73-Cre* allele drives recombination in dentate gyrus granule neurons in the adult hippocampus of multiple reporter lines

**Figure 4.**
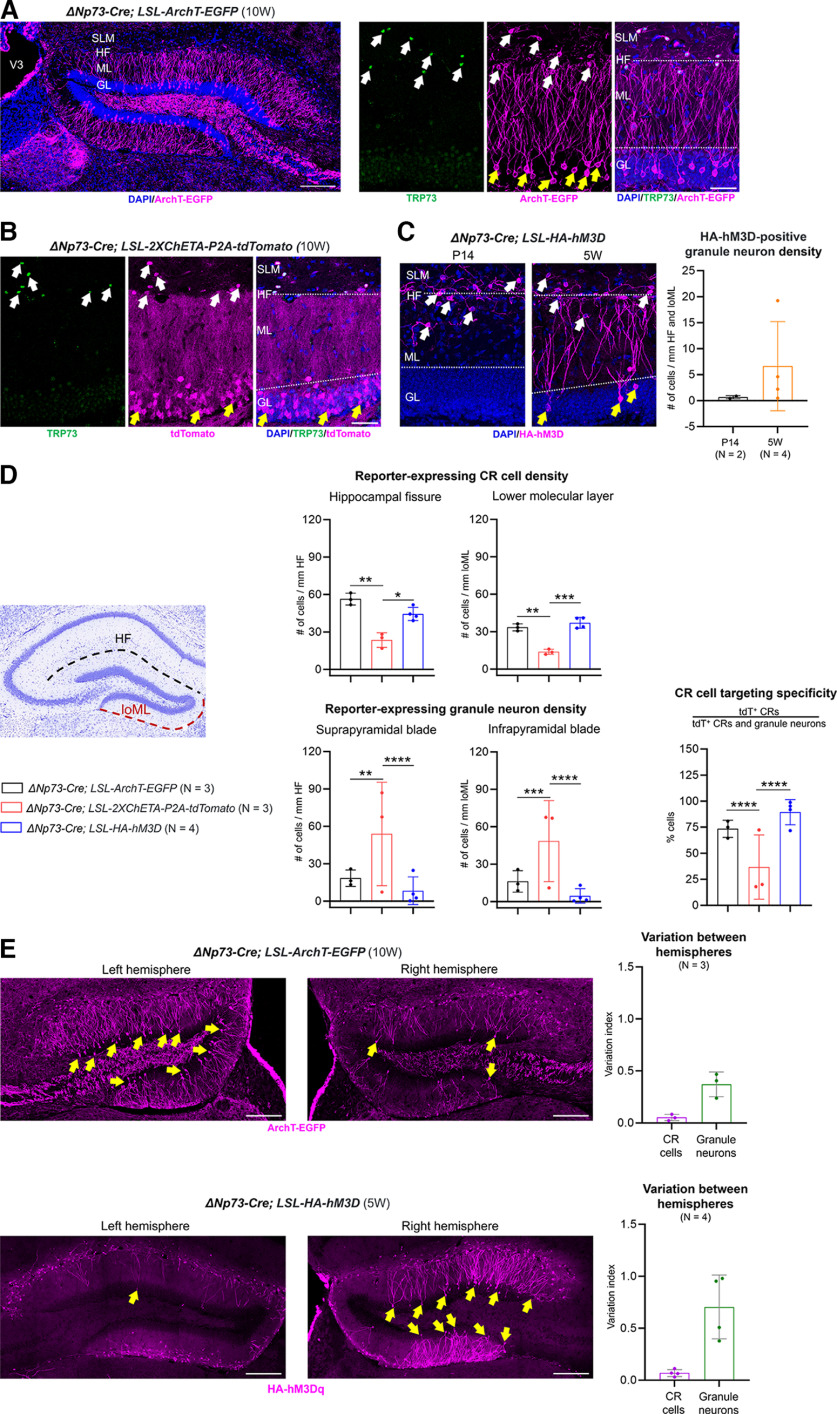
The Δ*Np73-Cre* allele drives reporter expression in Cajal–Retzius cells and adult dentate gyrus granule neurons in different reporter lines. ***A***, Left, Overview of ArchT-EGFP expression in the DG of 10-week-old Δ*Np73-Cre; LSL-ArchT-EGFP* mice. Note the extensive ArchT-EGFP fusion protein expression in the dendrites and axons of DG granule neurons. Scale bar, 200 μm. Right, Representative high-magnification images. ArchT-EGFP expression is found in the plasma membrane of TRP73^+^ CR cells (white arrows) and TRP73^–^ DG granule neurons (yellow arrows). Scale bar, 50 μm. ***B***, tdTomato reporter expression in the DG of 10-week-old (10W) Δ*Np73-Cre; LSL-2XChETA-P2A-tdTomato* mice. In these mice, ChETA and tdTomato are expressed separately and not as fusion proteins. tdTomato expression is found in the cytoplasm of TRP73^+^ CR cells (white arrows) and TRP73^–^ DG granule neurons (yellow arrows). Scale bar, 50 μm. ***C***, HA-hM3D reporter expression in the dentate gyrus of the Δ*Np73-Cre; LSL-HA-hM3D* mice. At P14, reporter expression is restricted to CR cells (white arrows). By 5 weeks of age, reporter expression is also observed in DG granule neurons (yellow arrows). Scale bar, 50 μm. Quantification of reporter-expressing granule neuron density is shown to the right. Cell densities were normalized to the length of the hippocampal fissure (HF) and the molecular layer of the DG infrapyramidal blade (loML). ***D***, Quantification of reporter-expressing CR cell and granule neuron densities, as well as CR cell-targeting specificity in the three mouse lines. ***E***, Representative confocal images of reporter expression in the two hemispheres of a Δ*Np73-Cre; LSL-ArchT-EGFP* mouse (top) and a Δ*Np73-Cre; LSL-HA-hM3D* mouse (bottom). Yellow arrows indicate reporter-expressing DG granule neurons. Scale bars, 200 μm. Quantification of variation indexes (the difference of cell densities between the two hemispheres divided by the sum of the cell densities) are shown to the right. V3, Third ventricle; SLM, stratum lacunosum-moleculare; GL, granular layer. Data are presented as scatter plots with all data points shown and error bars representing ±SD. Each data point is an individual animal, whereby three sections were measured for each animal. Statistical analyses were performed using nested *t* test or nested one-way ANOVA with Tukey’s *post hoc* test. *p <* *0.05; **p <* *0.01; ***p <* *0.001; ****p <* *0.0001. The images that show the lack of reporter expression in the hippocampus of the *LSL-tdTomato* mice are provided in Extended Data Figure 4-1. The data that demonstrate reporter expression in the neocortex are shown in Extended Data Figure 4-2.

While the *Wnt3a-Cre* allele has been shown to induce recombination in non-CR cells in the DG (Quattrocolo and Maccaferri, 2014; Anstötz et al., 2018a), the surprising degree of recombination in granule neurons by the Δ*Np73-Cre* allele has not been previously appreciated. We asked whether these recombination events could be because of the *LSL-tdTomato* reporter undergoing recombination in the absence of the Cre recombinase. However, we did not observe any tdTomato expression in granule neurons in the reporter-only mice (without the Cre allele; Extended Data Fig. 4-1). To further test this in a more rigorous way, we crossed the Δ*Np73-Cre* transgenic mice to two different reporter lines, *LSL-ArchT-EGFP* and *LSL-2XChETA-P2A-tdTomato*, expressing inhibitory and excitatory opsins, respectively, in a Cre-dependent manner. Adult (10 weeks old) Δ*Np73-Cre*; *LSL-ArchT-EGFP* (*ArchT*^Δ^*^Np73-Cre^*, for short) mice exhibited ArchT-EGFP fusion protein expression in CR cells, which were marked by the CR cell-specific protein TRP73. However, they also showed widespread EGFP expression in DG granule neurons (Fig. 4*A*). Similarly, we found reporter expression in both TRP73^+^ CR cells and TRP73^–^ DG granule neurons in adult Δ*Np73-Cre*; *LSL-2XChETA-P2A-tdTomato* (*ChETA*^Δ^*^Np73-Cre^*, for short) mice (Fig. 4*B*). All three of the aforementioned reporter alleles—the *LSL-tdTomato*, the *LSL-ArchT-EGFP*, and the *LSL-2XChETA-P2A-tdTomato*—were inserted into the *ROSA26* locus on chromosome 6, which is widely used to harbor reporter constructs because of its ubiquitous expression (Soriano, 1999). As genomic context may influence reporter expression, we next crossed the Δ*Np73-Cre* mice to the *LSL-HA-hM3D* mice, in which the Cre-dependent chemogenetic receptor (*hM3D*) cassette was integrated into chromosome 14 as a transgene (https://www.jax.org/strain/026220). At P14, we observed robust and specific reporter expression in neocortical and hippocampal CR cells in the Δ*Np73-Cre*; *LSL-HA-hM3D* (*hM3D*^Δ^*^Np73-Cre^*, for short) mice (Fig. 4*C*, Extended Data Fig. 4-2*C*). At this age, a low degree of recombination (<1 cell/mm) was seen in granule neurons, which increased to ∼6 cells/mm at 5 weeks of age (Fig. 4*C*), which is consistent with our findings in the Δ*Np73-Cre; LSL-tdTomato* mice (Fig. 2).

10.1523/ENEURO.0054-23.2023.f4-1 Figure 4-1 Absence of Cre-independent recombination events in the hippocampus of the *LSL-tdTomato*/^+^ heterozygous mice. Representative confocal images of immunostaining for tdTomato from three different animals are shown. A total of 4 animals (3 sections/animal) were examined. CA, Cornu ammonis. Scale bar, 200 μm. Download Figure 4-1, TIF file.

10.1523/ENEURO.0054-23.2023.f4-2 Figure 4-2 Reporter expression in neocortex layer 1 in different reporter lines. ***A***, Representative confocal images show expression of the ArchT-EGFP reporter in a CR cell (yellow arrows) in layer 1 of a 10-week-old Δ*Np73-Cre; LSL-ArchT-EGFP* mouse. Reporter expression was present only in CR cells. Scale bar, 50 μm. ***B***, Representative confocal images show the expression of the toTomato reporter in a CR cell in layer 1 of a 10-week-old Δ*Np73-Cre; LSL-2XChETA-P2A-tdTomato* mouse. Reporter expression was present not only in CR cells (yellow arrows) but also in non-CR cells in layer 1 (white yellowheads). Scale bar, 50 μm. ***C***, Representative confocal images show expression of the HA-hM3Dq reporter in CR cells (yellow arrows) in layer 1 of a P14 (top) and a 5-week-old (bottom) Δ*Np73-Cre; LSL-HA-hM3D* mouse. Reporter expression was restricted to CR cells. Scale bar, 50 μm. ***D***, Quantification of neocortical layer 1 reporter-expressing CR cell density of the three reporter lines. There was no significant difference between the groups. Data are presented as a scatter plot with all data points shown and error bars representing ±SD. Each data point is an individual animal, whereby three sections were measured for each animal. Statistical analysis was performed using nested one-way ANOVA with Tukey’s *post hoc* test. Download Figure 4-2, TIF file.

We further compared the on-target and off-target recombination events of the three reporter lines under the Δ*Np73-Cre* driver. To this end, we quantified the number of reporter-expressing CR cells along the hippocampal fissure and in the molecular layer of the DG infrapyramidal blade, as well as the number of reporter-expressing DG granule neurons in the suprapyramidal and infrapyramidal blades. The numbers of CR cells and granule neurons were similar between the *ArchT*^Δ^*^Np73-Cre^* and the *hM3D*^Δ^*^Np73-Cre^* mice (Fig. 4*D*). In contrast, the *ChETA*^Δ^*^Np73-Cre^* mice had a lower number of labeled CRs but a higher number of granule neurons, resulting in lower CR cell-targeting specificity. While all three reporter lines had a similar number of reporter-expressing CR cells in the adult neocortex (Extended Data Fig. 4-2*D*), recombination in the *ArchT*^Δ^*^Np73-Cre^* and the *hM3D*^Δ^*^Np73-Cre^* mice was restricted to CR cells in neocortex layer 1, while additional recombination was observed in non-CR cells in layer 1 in the *ChETA*^Δ^*^Np73-Cre^* mice (Extended Data Fig. 4-2*B*).

Similar to our findings with the Δ*Np73-Cre*; *LSL-tdTomato* mice (Fig. 3), the extent of Cre-mediated recombination in DG granule neurons was highly variable between individual *ChETA*^Δ^*^Np73-Cre^* mice (Fig. 4*D*, note the large variation among the three animals). Although this between-mice variation was less in the *ArchT*^Δ^*^Np73-Cre^* and the *hM3D*^Δ^*^Np73-Cre^* lines, recombination events in DG granule neurons varied substantially between the two hemispheres within a single mouse in these two lines (Fig. 4*E*). When we quantified this using a variation index, we found that the between-hemisphere variation indexes for CR cells were close to zero in both the *ArchT*^Δ^*^Np73-Cre^* and the *hM3D*^Δ^*^Np73-Cre^* mice, whereas the variation indexes for granule neurons were larger and more variable (Fig. 4*E*). Collectively, our data demonstrate that, while the degree and variability of recombination in granule neurons differ among reporter lines, recombination events in postnatal DG granule neurons driven by the Δ*Np73-Cre* allele is a consistent phenomenon among different reporter lines.

### Neonatal intraventricular injection of adeno-associated virus efficiently transduces Cajal–Retzius cells

**Figure 5.**
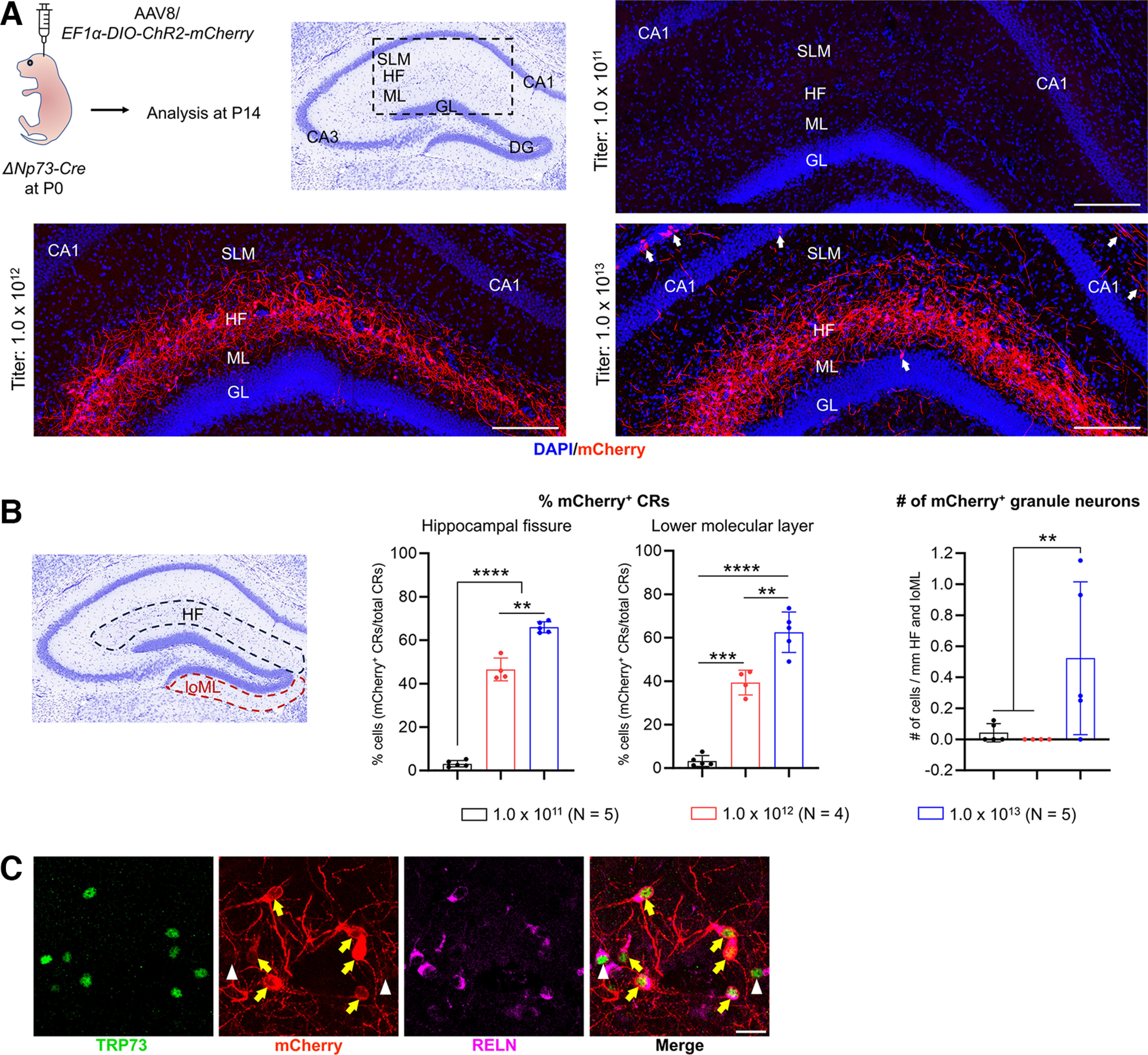
Neonatal intraventricular injection of adeno-associated virus induces efficient recombination events in hippocampal Cajal–Retzius cells in the Δ*Np73-Cre* mice. ***A***, Schematic shows the experimental procedure and brain region of interest. The lateral ventricles of P0 Δ*Np73-Cre* mouse pups were freehand injected with different titers of AAV serotype 8 carrying a Cre-dependent expression construct for the ChR2-mCherry fusion protein under the *EF1α* promoter (AAV8/*EF1α-DIO-ChR2-mCherry*). Injected mice were analyzed at P14. Three representative confocal images show the hippocampal fissure (HF) area after intraventricular injection of three titers of the AAV8 for comparison of transduction efficiency. Viral-mediated, Cre-dependent transgene expression is visualized by immunostaining for mCherry. Note the lack of transduction using the 1.0 × 10^11^ viral titer. Cre-independent spontaneous recombination events were observed in some cortical and hippocampal neurons in mice injected with the 1.0 × 10^13^ titer (white arrows). Scale bars, 200 μm. ***B***, Quantification of viral transduction efficiency in CR cells and recombination events in granule neurons. Data are presented as scatter plots with all data points shown and error bars representing ±SD. Each data point is an individual animal, whereby three sections were measured for each animal. The total number of CR cells per animal analyzed along the hippocampal fissure of different animals were as follows: 1.0 × 10^11^ titer, 90–174 cells; 1.0 × 10^12^ titer, 82–152 cells; 1.0 × 10^13^ titer, 108–188 cells. The total number of CR cells per animal analyzed in the molecular layer of the infrapyramidal blade of different animals were as follows: 1.0 × 10^11^ titer, 47–83 cells; 1.0 × 10^12^ titer, 39–82 cells; 1.0 × 10^13^ titer, 42–77 cells. Statistical analyses were performed using nested one-way ANOVA with Tukey’s *post hoc* test. **p <* *0.01; ***p <* *0.001; ****p <* *0.0001. ***C***, Immunostaining for TRP73, mCherry, and RELN in P14 1.0 × 10^12^ GC/ml virus-injected tissue confirmed that mCherry-expressing cells (yellow arrows) are CR cells. Some CR cells are not transduced as they do not express mCherry (white arrowheads). Scale bar, 20 μm. GL, Granular layer; ML, molecular layer; SLM, stratum lacunosum-moleculare; CA1, Cornu Ammonis 1; CA3, Cornu Ammonis 3; loML, lower molecular layer (i.e., molecular layer of the infrapyramidal blade of the dentate gyrus). The images that illustrate the result using AAV/*CAG-YFP* are shown in Extended Data Figure 5-1. The images that show the specificity of our neonatal AAV injection approach in the hippocampus are provided in Extended Data Figure 5-2. The data that demonstrate the absence of Cre-mediated recombination in the wild-type mice are shown in Extended Data Figure 5-3.

The unexpected recombination pattern driven by the Δ*Np73-Cre* could confound the interpretation of experiments involving genetic crosses to floxed alleles. We therefore set out to develop a strategy for efficient and specific genetic manipulation of CR cells. This was guided by our observation that, before the first two postnatal weeks, recombination by the Δ*Np73-Cre* allele in the hippocampus was highly specific to CR cells (Figs. 2*A*, 4*C*). Imposing additional specificity that limits recombination in DG neurons that are born after P14 could be a viable approach. Fortuitously, neonatal (P0) intracerebroventricular injection of AAV has been shown to produce very little viral-mediated expression in DG granule neurons, especially those generated after P14 (Kim et al., 2013, 2014). This freehand injection method is fast and easy to use. By opting for a serotype such as AAV8, it results in brain-wide transduction (Kim et al., 2013), which is particularly desirable for CR cells as they are distributed throughout the hippocampus. We first conducted a pilot experiment by injecting AAV8 carrying a viral construct encoding YFP under the CAG promoter (AAV8/*CAG-YFP*) into P0 wild-type mice and analyzed them at P8 (Extended Data Fig. 5-1*A*). We found that the AAV8 serotype effectively transduced TRP73^+^ CR cells (Extended Data Fig. 5-1*B*, yellow arrows). We next asked whether we could use neonatal intracerebroventricular injection of Cre-dependent AAV8 constructs to impose additional temporal specificity in the Δ*Np73-Cre* mice. To test this, we injected P0 Δ*Np73-Cre* pups intraventricularly with AAV8 carrying a Cre-dependent expression construct for the ChR2-mCherry fusion protein driven by the ubiquitous EF1α promoter [AAV8/*EF1α-DIO-ChR2-mCherry* (or AAV/*DIO-ChR2* for short); Fig. 5*A*]. As the viral titer may impact the efficiency and specificity of transduction, we determined the optimal titer by assessing transduction efficiency and specificity at 1.0 × 10^11^, 1.0 × 10^12^, and 1.0 × 10^13^ genome copies (GC)/ml. At 14 d post injection (which is also P14), we found very few CR cells with mCherry expression in mice injected with 1.0 × 10^11^ GC/ml virus (Fig. 5*A*,*B*), indicating that the viral titer was too low to render detectable transduction. In contrast, Δ*Np73-Cre* mice injected with 1.0 × 10^12^ GC/ml AAV8 showed robust reporter expression in ∼45% of CR cells along the hippocampal fissure and ∼40% of CR cells in the molecular layer of the infrapyramidal blade of the dentate gyrus (lower molecular layer). The identity of mCherry-expressing CR cells was further confirmed by immunostaining for the CR cell markers TRP73 and RELN (Fig. 5*C*). With this titer, viral transduction was restricted to CR cells in the hippocampus, as we did not observe reporter expression in any other hippocampal neurons such as DG granule neurons (Fig. 5*A*,*B*, Extended Data Fig. 5-2*A*). Slightly but significantly more robust CR cell transduction was observed along the hippocampal fissure (∼65%) and in the lower molecular layer (∼63%) in Δ*Np73-Cre* pups injected with 1.0 × 10^13^ GC/ml virus. However, at such a high titer, we began to see occasional reporter expression in DG granule neurons (∼1–3 cells/section; Fig. 5*B*, cell density calculation) and in CA1 neurons as well as other cortical neurons (Fig. 5*A*, white arrows). This could be because of Cre-independent spontaneous recombination of the viral vector (Fischer et al., 2019; Botterill et al., 2021). To test this, we injected wild-type mice with AAV/*DIO-ChR2* at a titer of 1.0 × 10^13^ GC/ml and analyzed them at P14. Our data show that recombination events in the wild-type mice, which were Cre independent, were significantly fewer than those in the Δ*Np73-Cre* mice, which could be both Cre dependent and Cre independent (Extended Data Fig. 5-3*A*,*C*,*D*). This suggests that both Cre-dependent and Cre-independent recombination events may occur when viral titers are used at 1.0 × 10^13^ GC/ml. Additionally, two of the five pups injected with 1.0 × 10^13^ GC/ml virus were runty and reached humane endpoint by P14, while animals injected with lower titers appeared healthy until at least 7 weeks of age. Therefore, we opted to use the 1.0 × 10^12^ GC/ml titer of the virus for the remainder of the study. Overall, our data demonstrate the specificity and effectiveness of neonatal intracerebroventricular injections of AAVs in transducing CR cells in the hippocampus.

10.1523/ENEURO.0054-23.2023.f5-1 Figure 5-1 Adeno-associated virus serotype 8 efficiently transduces Cajal–Retzius cells when injected intraventricularly at postnatal day 0. ***A***, Schematic shows the intraventricular injection and brain region of interest. Animals were injected at P0 with AAV8 carrying a YFP expression construct driven by the *CAG* promoter (*CAG-YFP*), and brain tissue was analyzed at P8. ***B***, Many TRP73^+^ Cajal–Retzius cells express YFP (yellow arrows), while some do not (white arrows). Some granule neurons in the granular layer (GL, white arrowheads) also express YFP. HF, Hippocampal fissure; ML, molecular layer; GL, granular layer. Scale bar, 20 μm. Download Figure 5-1, TIF file.

10.1523/ENEURO.0054-23.2023.f5-2 Figure 5-2 Neonatal intraventricular injection of Cre-dependent adeno-associated virus confers specificity in Cajal–Retzius cells. ***A***, ***B***, Neonatal (P0) Δ*Np73-Cre* pups were injected with 1.0 × 10^12^ GC/mL AAV8/*EF1α-DIO-ChR2-mCherry* (***A***) or AAV8/*hSyn-DIO-hM3D-mCherry* (***B***), and brain tissue analyzed at P14 or 7 weeks of age. CA, Cornu Ammonis. Scale bar, 300 μm. Download Figure 5-2, TIF file.

10.1523/ENEURO.0054-23.2023.f5-3 Figure 5-3 Absence of Cre-mediated recombination in Cajal–Retzius cells in wild-type mice neonatally injected with AAV8/*EF1a-DIO-ChR2-mCherry*. ***A***, ***B***, Wild-type pups (littermates of Δ*Np73-Cre* mice) injected at P0 with AAV8/*EF1a-DIO-ChR2-mCherry* were analyzed for mCherry expression at 14 days (P14; ***A***) or 7 weeks (***B***) postinjection. Coimmunostaining of TRP73 and RELN identifies CR cells (yellow arrows), which do not express mCherry. Scale bars: top, 200 μm; bottom, 20 μm. SLM, Stratum lacunosum-moleculare; HF, hippocampal fissure; ML, molecular layer; GL, granular layer; CA, cornu ammonis. ***C***, Quantification of the proportion of mCherry^+^ CR cells and the density of mCherry^+^ granule neurons. ***D***, Quantification of CR cell-targeting specificity in P14 wild-type and Δ*Np73-Cre* mice injected with AAV8/*EF1a-DIO-ChR2-mCherry* at P0. Data are presented as scatter plots with all data points shown and error bars representing ±SD, and statistical analyses were performed using nested a *t* test or one-way ANOVA with Tukey’s *post hoc* test. Each data point is an individual animal, whereby three sections were measured for each animal. *p < 0.05. Download Figure 5-3, TIF file.

### Neonatal intraventricular injection of Cre-dependent adeno-associated virus confers specificity in Cajal–Retzius cells in the adult hippocampus

**Figure 6.**
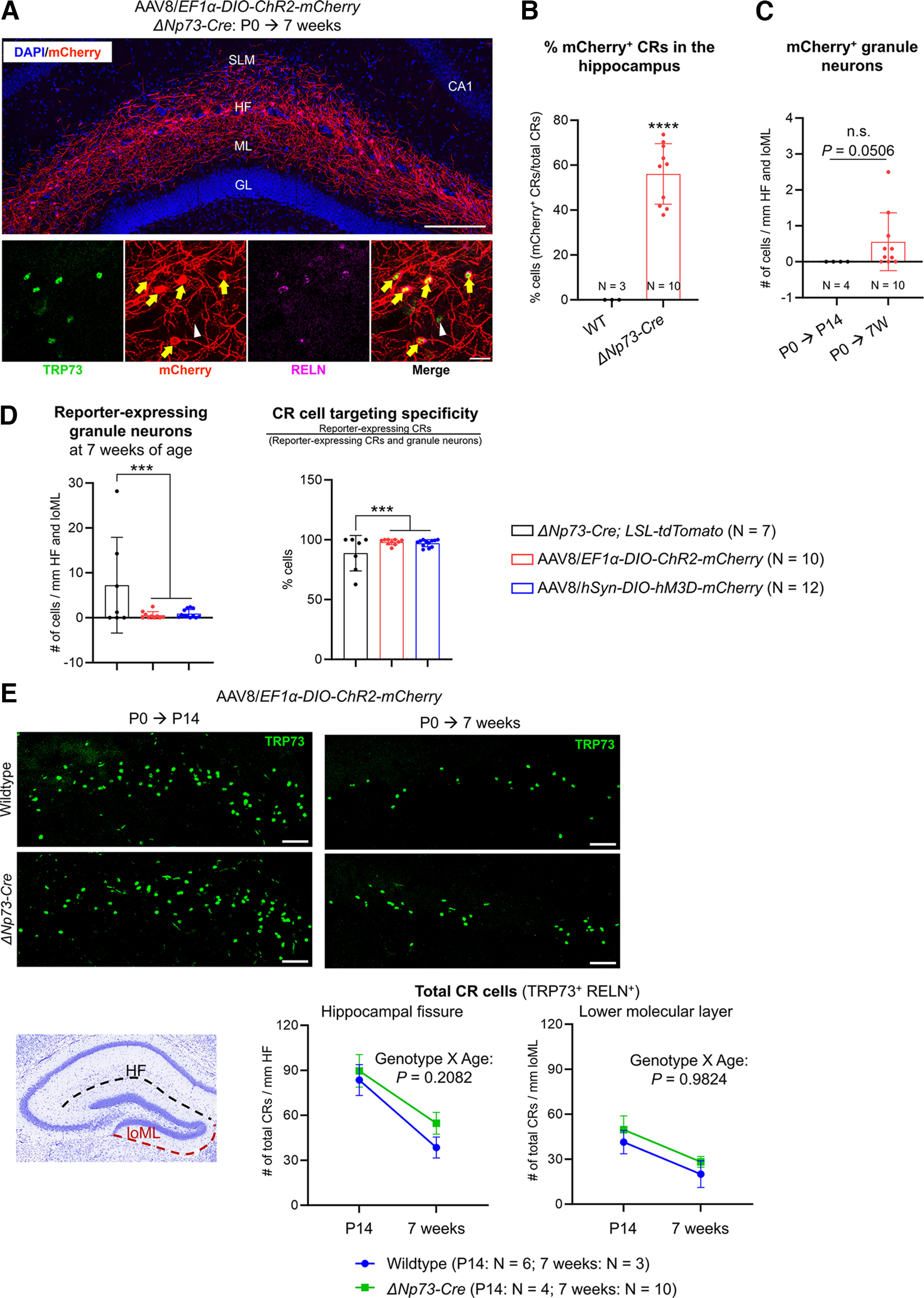
Neonatal intraventricular injection of adeno-associated virus results in lasting and highly specific transgene expression in Cajal–Retzius cells. ***A***, Representative images of a Δ*Np73-Cre* mouse hippocampus 7 weeks post-neonatal (P0) injection with AAV8/*EF1a-DIO-ChR2-mCherry*. Coimmunostaining with TRP73 and RELN indicates CR cells continue to express mCherry 7 weeks following the injection (yellow arrows). Some CR cells do not express mCherry (white arrowhead) because they were not transduced by the virus. Scale bars: top, 200 μm; bottom, 20 μm. ***B***, Quantification of AAV-transduced CR cells (mCherry^+^ TRP73^+^) in 7-week-old wild-type (WT) and Δ*Np73-Cre* mice. ***C***, Comparison of the number of mCherry^+^ granule neurons in P14 and 7-week-old Δ*Np73-Cre* mice neonatally injected with AAV8/*EF1a-DIO-ChR2-mCherry*. ***D***, Comparison of the number of recombination events in granule neurons and CR cell-targeting specificity of 7-week-old Δ*Np73-Cre; LSL-tdTomato* mice, Δ*Np73-Cre* mice neonatally injected with AAV8/*EF1a-DIO-ChR2-mCherry*, and Δ*Np73-Cre* mice neonatally injected with AAV8/*hSyn-DIO-hM3D-mCherry*. ***E***, Representative images of the hippocampal fissure areas of P14 and 7-week-old wild-type and Δ*Np73-Cre* transgenic mice injected neonatally with AAV8/*EF1a-DIO-ChR2-mCherry*. Scale bars, 20 μm. Quantification of the densities of TRP73^+^ CR cells are shown at the bottom. SLM, Stratum lacunosum-moleculare; HF, hippocampal fissure; ML, molecular layer; GL, granular layer; CA1, Cornu Ammonis 1; loML, lower molecular layer (i.e., molecular layer of the infrapyramidal blade of the dentate gyrus). Data are presented as scatter plots in ***B***, ***C***, and ***D*** with all data points shown and error bars representing ±SD, and statistical analyses were performed using nested one-way ANOVA with Tukey’s *post hoc* test. Each data point is an individual animal, whereby three sections were measured for each animal. Data are presented as summary data in ***E*** with mean ± SD, and statistical analyses were performed using two-way ANOVA with Sidak test to correct for multiple comparisons. ***p <* *0.001; ****p <* *0.0001; n.s., not significant.

We next assessed whether neonatal intracerebroventricular injections of Cre-dependent AAV could impose sufficient temporal specificity that overcomes excessive recombination in postnatal DG granule neurons in the Δ*Np73-Cre* mice. To this end, animals were injected intraventricularly at P0 with 1.0 × 10^12^ GC/ml AAV8/*DIO-ChR2* and allowed to age to 7 weeks of age. Our analysis showed that ∼56% of CR cells expressed the mCherry reporter at this age (Fig. 6*A*,*B*, Extended Data Fig. 5-3*B*,*C*), confirming robust viral transduction. Importantly, recombination events in DG granule neurons were limited: of the 10 animals that we examined, 3 exhibited no recombination in granule neurons, while the other 7 animals had a limited number of recombined granule neurons (Fig. 6*C*). Compared with similarly injected mice at P14, there was a trend toward more recombined granule neurons at 7 weeks of age, but this did not reach significance. Nonetheless, in 7-week-old Δ*Np73-Cre* mice injected with AAV8/*DIO-ChR2* at P0, the number of granule neurons with recombination events is significantly less than that in 7-week-old Δ*Np73-Cre; LSL-tdTomato* mice, resulting in higher CR cell-targeting specificity (Fig. 6*D*). This demonstrates that our approach of neonatal intracerebroventricular AAV injection into the Δ*Np73-Cre* pups effectively limits recombination events in granule neurons in adult mice.

CR cells are known to undergo massive cell death in early postnatal weeks (Causeret et al., 2021). We next assessed whether viral transduction or transgene expression would alter the developmental programmed cell death of CR cells by comparing wild-type and Δ*Np73-Cre* mice injected with the AAV. We analyzed the numbers of TRP73^+^ RELN^+^ CR cells (total CR cells) along the hippocampal fissure and in the lower molecular layer at P14, during the time window of massive CR cell death and at 7 weeks of age, when CR cell death has subsided (Anstötz et al., 2018a). We did not find a significant genotype and age interaction in our analyses for CR cells in either area (Fig. 6*E*). These data suggest that AAV transduction does not significantly alter physiological programmed cell death of CR cells. Altogether, our data validate the utility of neonatal intracerebroventricular injection of Cre-dependent AAV as a tool to specifically manipulate CR cells in the adult hippocampus with minimal recombination in DG granule neurons.

### Neonatal intraventricular injection of adeno-associated virus facilitates neural activity modulation of Cajal–Retzius cells in the adult hippocampus

**Figure 7.**
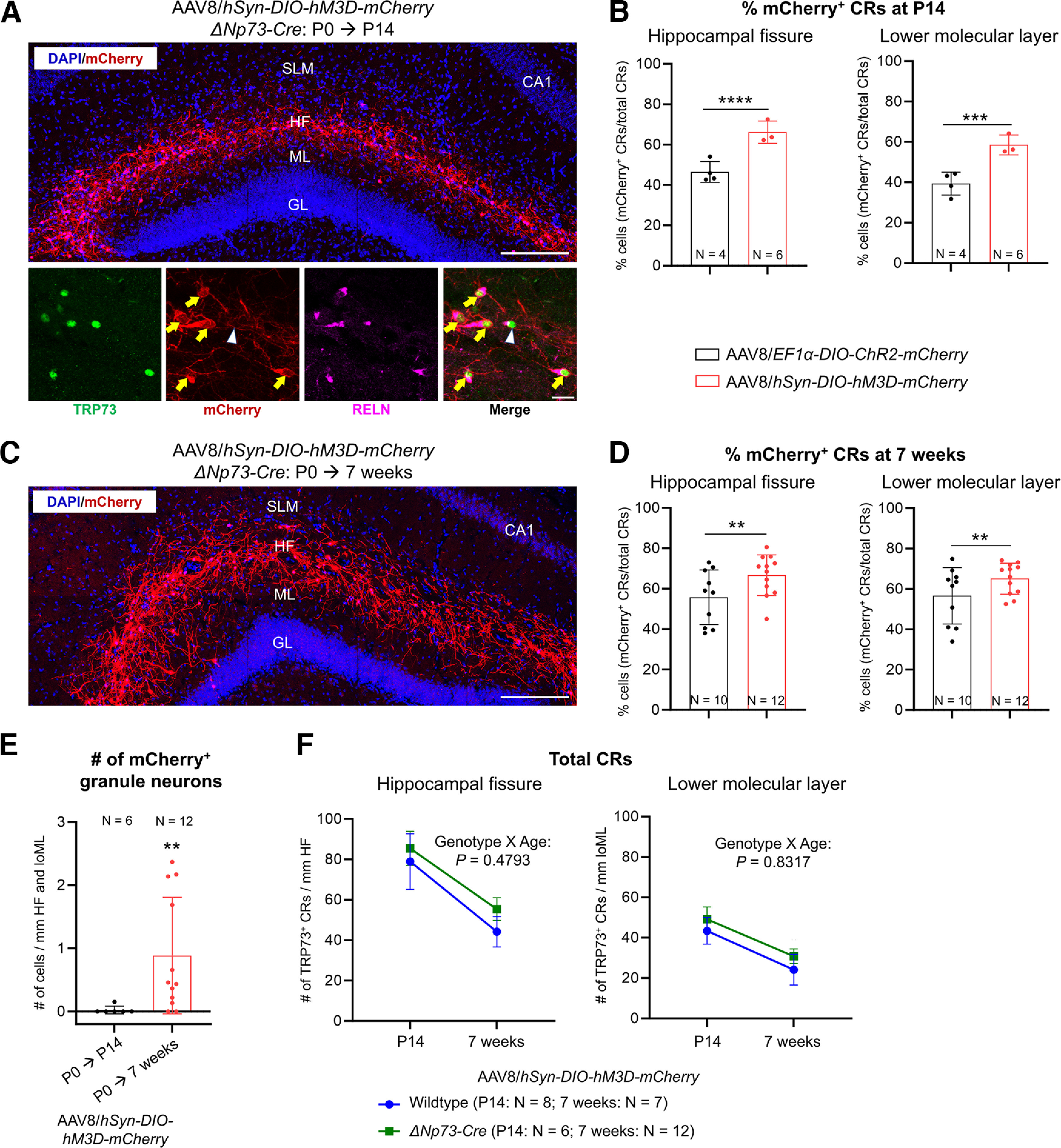
Neonatal intraventricular injection of AAV8/*hSyn-DIO-hM3D-mCherry* results in robust and highly specific transgene expression in Cajal–Retzius cells. ***A***, Neonatal Δ*Np73-Cre* pups were injected with adeno-associated virus serotype 8 carrying a Cre-dependent expression construct for the hM3D-mCherry fusion protein under the *hSyn* promoter (AAV8/*hSyn-DIO-hM3D-mCherry*) and brain tissue analyzed at P14. mCherry-expressing cells also express TRP73 and RELN, confirming their identity as CR cells (yellow arrows). Some CR cells do not express mCherry as they were not transduced by the injected AAV8 (white arrowhead). Scale bars: top, 200 μm; bottom, 20 μm. ***B***, Comparison of viral transduction efficiency in CR cells at P14 of the two different viral constructs (AAV8/*EF1α-DIO-ChR2-mCherry* and AAV8/*hSyn-DIO-hM3D-mCherry*). ***C***, Reporter expression persists for 7 weeks after neonatal injection. A representative image of a Δ*Np73-Cre* mouse hippocampus 7 weeks post injection with AAV8/*hSyn-DIO-hM3D-mCherry* is shown. Scale bar, 200 μm. ***D***, Comparison of viral transduction efficiency in CR cells at 7 weeks of age of the two different viral constructs (AAV8/*EF1α-DIO-ChR2-mCherry* and AAV8/*hSyn-DIO-hM3D-mCherry*). ***E***, Comparison of the number of mCherry^+^ granule neurons in P14 and 7-week-old Δ*Np73-Cre* mice neonatally injected with AAV8/*hSyn-DIO-hM3D-mCherry*. ***F***, Quantification of the densities of TRP73^+^ CR cells in P14 and 7-week-old wild-type or Δ*Np73-Cre* mice neonatally injected with AAV8/*hSyn-DIO-hM3D-mCherry*. SLM, Stratum lacunosum-moleculare; HF, hippocampal fissure; ML, molecular layer; GL, granular layer; CA1, Cornu Ammonis 1; loML, lower molecular layer (i.e., molecular layer of the infrapyramidal blade of the dentate gyrus). Data are presented in ***B***, ***D***, and ***E*** as scatter plots with all data points shown and error bars representing ±SD; statistical analyses were performed using nested *t* tests. Each data point is an individual animal, whereby three sections were measured for each animal. Data are presented as summary data in ***F*** with mean ± SD, and statistical analyses were performed using two-way ANOVA with Sidak test to correct for multiple comparisons. **p <* *0.01; ***p <* *0.001; ****p <* *0.0001. The data that demonstrate the absence of Cre-mediated recombination in the wild-type mice are shown in Extended Data Figure 7-1. Analyses of the effect of sex on AAV transduction efficiency are provided in Extended Data Figure 7-2.

One potential application for specific genetic manipulation in adult CR cells is to manipulate their neural activity and determine the effects on neural circuits and animal behavior. We therefore undertook a proof-of-principle study to determine whether neonatal intracerebroventricular AAV injection into the Δ*Np73-Cre* mice is suitable for this application. Neural activity modulation may be achieved using either optogenetics or chemogenetics. While optogenetics has a localized effect because of the limits of light delivery, chemogenetics is often used for broad or brain-wide targeting (Vlasov et al., 2018). As CR cells are widely distributed throughout the adult hippocampus, we decided to use chemogenetics to activate these cells. To this end, Δ*Np73-Cre* P0 pups were injected intraventricularly with 1.0 × 10^12^ GC/ml AAV8 carrying a Cre-dependent expression construct of hM3D(Gq)-mCherry fusion protein driven by the neuronal promoter *hSyn* [AAV8/*hSyn-DIO-hM3D-mCherry* (or AAV8/*DIO-hM3D* for short)]. At P14, ∼66% of CR cells along the hippocampal fissure and ∼59% of CR cells in the lower molecular layer expressed the mCherry reporter (Fig. 7*A*,*B*, Extended Data Fig. 7-1*A*). Robust mCherry reporter expression persisted in 7-week-old Δ*Np73-Cre* mice injected neonatally with AAV8/*DIO-hM3D* (Fig. 7*C*,*D*, Extended Data Figs. 5-2*B*, 7-1*B*). Compared with our results using the AAV8/*DIO-ChR2*, the AAV8/*DIO-hM3D* virus transduced a significantly higher proportion of CR cells at both ages examined (Fig. 7*B*,*D*, Extended Data Fig. 7-2), suggesting that the *hSyn* promoter may be more robust than the *EF1α* promoter for driving transgene expression in hippocampal CR cells.

10.1523/ENEURO.0054-23.2023.f7-1 Figure 7-1 Absence of Cre-mediated recombination in Cajal–Retzius cells in wild-type mice neonatally injected with AAV8/*hSyn-DIO-hM3D-mCherry*. ***A***, ***B***, Wild-type pups (littermates of Δ*Np73-Cre* mice) injected at P0 with AAV8/*hSyn-DIO-hM3D-mCherry* were analyzed for mCherry expression at 14 days (P14; ***A***) or 7 weeks (***B***) postinjection. Immunostaining of TRP73 identifies Cajal–Retzius cells (yellow arrows), which do not express mCherry. Some mCherry expression was observed in processes (white arrowheads) that do not belong to Cajal–Retzius cells. These were likely results of Cre-independent spontaneous recombination events of the viral vector during virus preparation, which is a well documented phenomenon for the double-floxed inverse orientation (DIO) system. SLM, Stratum lacunosum-moleculare; HF, hippocampal fissure; ML, molecular layer; GL, granular layer; CA, cornu ammonis. Scale bars: top, 200 μm; bottom, 20 μm. ***C***, Quantification of the proportion of mCherry^+^ CR cells and the density of mCherry^+^ granule neurons. Data are presented as scatter plots with all data points shown and error bars representing ±SD, and statistical analyses were performed using nested one-way ANOVA with Tukey’s *post hoc* test. Each data point is an individual animal, whereby three sections were measured for each animal. Download Figure 7-1, TIF file.

10.1523/ENEURO.0054-23.2023.f7-2 Figure 7-2 Analysis of potential sex effects on 7-week-old Δ*Np73-Cre* mice neonatally injected with AAV8/*EF1α-DIO-ChR2-mCherry* or AAV8/*hSyn-DIO-hM3D-mCherry*. In male mice, AAV8/*hSyn-DIO-hM3D-mCherry* transduced Cajal–Retzius cells along the hippocampal fissure more efficiently than AAV8/*EF1α-DIO-ChR2-mCherry* did. Data are presented as scatter plots with all data points shown. Each data point is an individual animal, whereby three sections were measured for each animal. Statistical analyses were performed using nested one-way ANOVA with Tukey’s *post hoc* test. **p <* *0.01; n.s., not significant. Download Figure 7-2, TIF file.

At P14, only one of the six Δ*Np73-Cre* mice injected with AAV8/*DIO-hM3D* showed recombination in DG granule neurons (one cell in one of three sections examined). This became more prevalent in mice at 7 weeks of age (Fig. 7*E*), indicating that recombination in granule neurons still occurred using our approach. However, similar to our findings using the AAV8/*DIO-ChR2*, the number of recombined granule neurons in 7-week-old AAV8/*DIO-hM3D*-injected mice is significantly lower, while the CR cell-targeting specificity is significantly higher, than that in the Δ*Np73-Cre; LSL-tdTomato* mice of the same age (Fig. 6*D*), again demonstrating that neonatal intracerebroventricular AAV injection into the Δ*Np73-Cre* pups effectively limits recombination events in granule neurons even in adult mice. We further determined that AAV8/*DIO-hM3D* transduction did not significantly alter the physiological cell death dynamics of CR cells (Fig. 7*F*).

**Figure 8.**
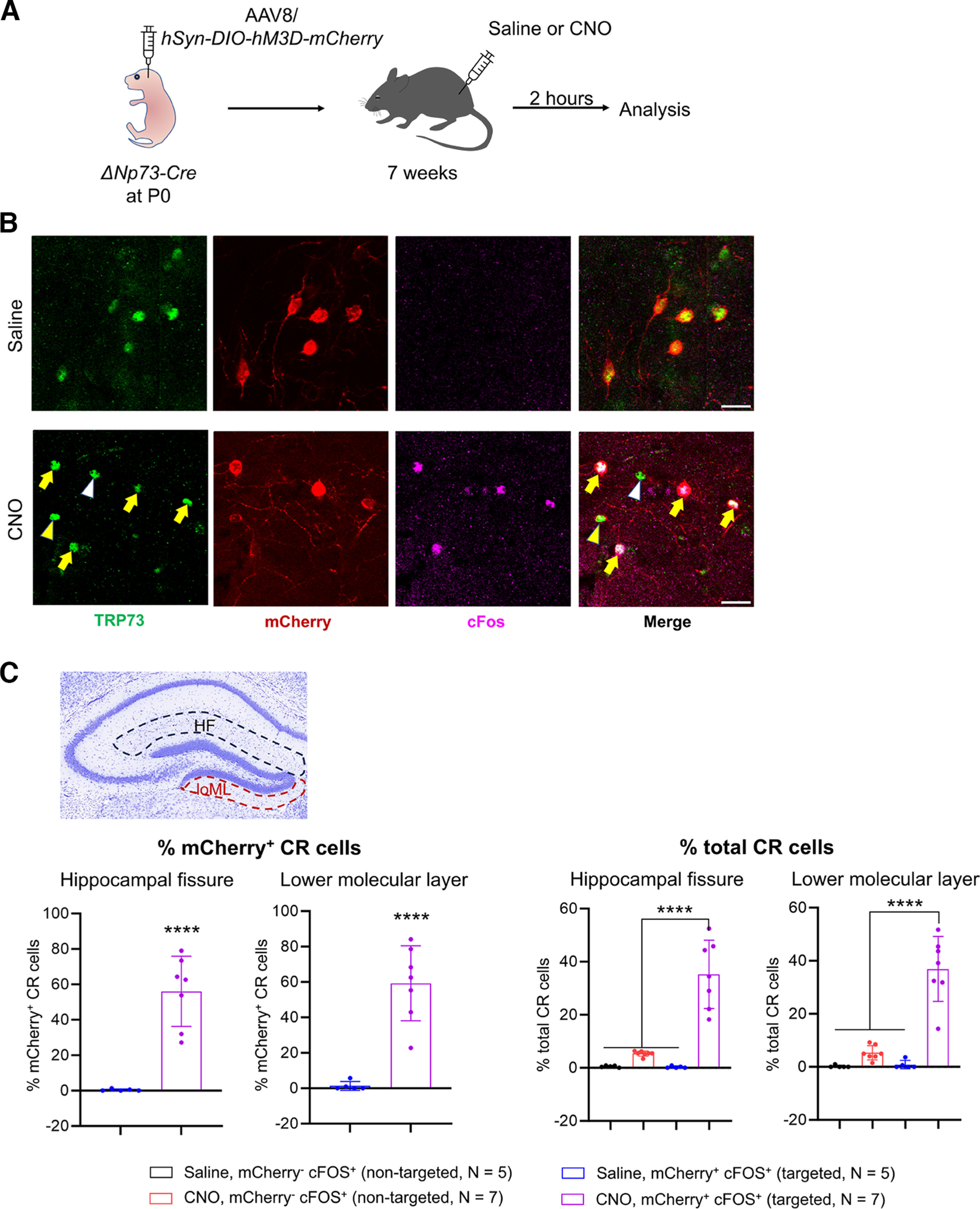
Chemogenetic activation of Cajal–Retzius cells in the adult hippocampus. ***A***, Schematic of the experimental procedures. ***B***, In 7-week-old mice neonatally injected with AAV8/*hSyn-DIO-hM3D-mCherry*, treatment of CNO results in activation of TRP73^+^ CR cells that express the chemogenetic receptor hM3D-mCherry, indicated by c-Fos immunoreactivity (yellow arrows). A CR cell with hM3D-mCherry expression but is not activated (mCherry^+^, c-Fos^–^) is indicated by the yellow arrowhead. A CR cell that does not express the hM3D receptor and is not positive for c-Fos is indicated by the white arrowhead. Scale bars, 20 μm. ***C***, Quantification of the percentage of different populations of c-Fos^+^ CR cells of mCherry^+^ or total CR cells. In each of the saline-injected control mice, 69–91 total CR cells were analyzed along the hippocampal fissure and 37–43 total CR cells were analyzed in the lower molecular layer. In each of the CNO-injected mice, 74–91 total CR cells were analyzed along the hippocampal fissure and 39–46 total CR cells were analyzed in the lower molecular layer. Data are presented as scatter plots with all data points shown and error bars representing ±SD. Each data point is an individual animal, whereby three sections were measured for each animal. Statistical analyses were performed using nested *t* test or nested one-way ANOVA with Tukey’s *post hoc* test. ****p <* *0.0001. The data that demonstrate the absence of CR cell activation in the wild-type mice are shown in Extended Data Figure 8-1.

To test the potential application of activity modulation in CR cells, we intraperitoneally injected CNO to activate the hM3D receptors (Fig. 8*A*), which leads to G-protein-coupled receptor signaling, intracellular calcium release, and neuronal excitation (Armbruster et al., 2007). We quantified the percentage of CR cells with c-Fos expression as a surrogate for neural activity (Fig. 8*B*). In saline-injected controls, we did not find any c-Fos expression in nontargeted (i.e., hM3D-mCherry^–^) CR cells, while only a few targeted (i.e., hM3D-mCherry^+^) CR cells exhibited c-Fos expression (Fig. 8*C*, Extended Data Fig. 8-1). This suggests that adult hippocampal CR cells were inactive under the specific conditions of our experiment and that CR cells expressing hM3D are not activated without CNO. Indeed, CNO administration led to a dramatic increase in the number of hM3D-mCherry^+^ CR cells with c-Fos immunoreactivity, with 55–60% hM3D-mCherry-expressing CR cells, which was ∼35% of the total CR cells, being activated along the hippocampal fissure and in the lower molecular layer (Fig. 8*C*). Our data thus provide proof-of-principle support for neonatal intracerebroventricular AAV injection into Δ*Np73-Cre* mice as a strategy to manipulate CR cell activity in the adult hippocampus.

10.1523/ENEURO.0054-23.2023.f8-1 Figure 8-1 Cajal–Retzius cells in wild-type mice neonatally injected with the AAV8/*hSyn-DIO-hM3D-mCherry* are not activated by the chemogenetics approach. Top, Schematic of the experimental approach. Bottom, Quantification of the different groups of CR cells. Few CR cells were c-Fos^+^ in the wild-type mice regardless of treatment with saline or CNO. HF, Hippocampal fissure; loML, lower molecular layer (i.e., molecular layer of the infrapyramidal blade of the dentate gyrus). Data are presented as scatter plots with all data points shown and error bars representing ±SD. Each data point is an individual animal, whereby three sections were measured for each animal. Statistical analyses were performed using nested one-way ANOVA with Tukey’s *post hoc* test. Download Figure 8-1, TIF file.

## Discussion

CR cells are important organizers of cortical development in the embryonic brain, but their persistence in the adult brain, especially the hippocampus, has largely been ignored (Anstötz et al., 2018a; Causeret et al., 2021). This conceptual bias, adding to the lack of suitable technical tools to specifically manipulate adult CR cells, contributes to the paucity of information on their function in the adult brain circuits and their influence on behavior. In this study, we show surprisingly high levels of recombination events in postnatal DG granule neurons in the Δ*Np73-Cre* genetic crosses to reporter lines. Seeking to restrict Cre-mediated recombination to CR cells, we develop a strategy that harnesses the efficiency of neonatal intracerebroventricular injection of viral vectors and the temporary specificity of the Δ*Np73-Cre* driver line. Our method is easy to implement, efficient, and versatile, offering a starting point for a wide range of experiments into CR cell function in the postnatal and adult hippocampus.

Our genetic crosses of the Δ*Np73-Cre* driver to reporter lines reveal unexpected recombination events in postnatal DG granule neurons. Because these recombination events vary substantially between mice and even within a single mouse, they are likely spontaneous events, rather than the Δ*Np73* promoter being turned on when DG granule neurons become mature. However, we cannot rule out the possibility that the Δ*Np73* promoter becomes active because of certain neural activity or dynamic cellular events during postnatal and/or adult hippocampal neurogenesis, resulting in Cre expression and recombination. Future studies using more animals could examine the effects of sex, housing conditions, handling, and animal behaviors on Δ*Np73-Cre*-mediated recombination in granule neurons. Nonetheless, our findings highlight the need for careful assessment of unwanted recombination events in genetic crosses of Cre driver lines to ensure that experimental results are generated from the intended genetic manipulation. Whether recombination outside of CR cells will confound result interpretation should be evaluated on a case-by-case basis with consideration of the potential contribution of each cell type. While lineage-tracing studies might tolerate erroneous recombination, where additional protein markers and morphologic and/or location information are available to aid in the interpretation of results, studies involving circuit manipulation or animal behavior may be less forgiving. For example, neural activity manipulation in adult Δ*Np73-Cre* mice that harbor opsins or chemogenetic receptors through genetic crosses will inevitably activate both CR cells and DG granule neurons. As CR cells modulate the hippocampal microcircuits (Quattrocolo and Maccaferri, 2014; Anstötz et al., 2016, 2018b, 2022) and DG granule neurons control hippocampal information processing (Jonas and Lisman, 2014), it may be difficult to tease apart the relative contributions of the two cells types if both are manipulated simultaneously. However, with our neonatal AAV injection approach, it is now possible to selectively activate or silence adult hippocampal CRs with limited effects on granule neurons.

**Figure 9.**
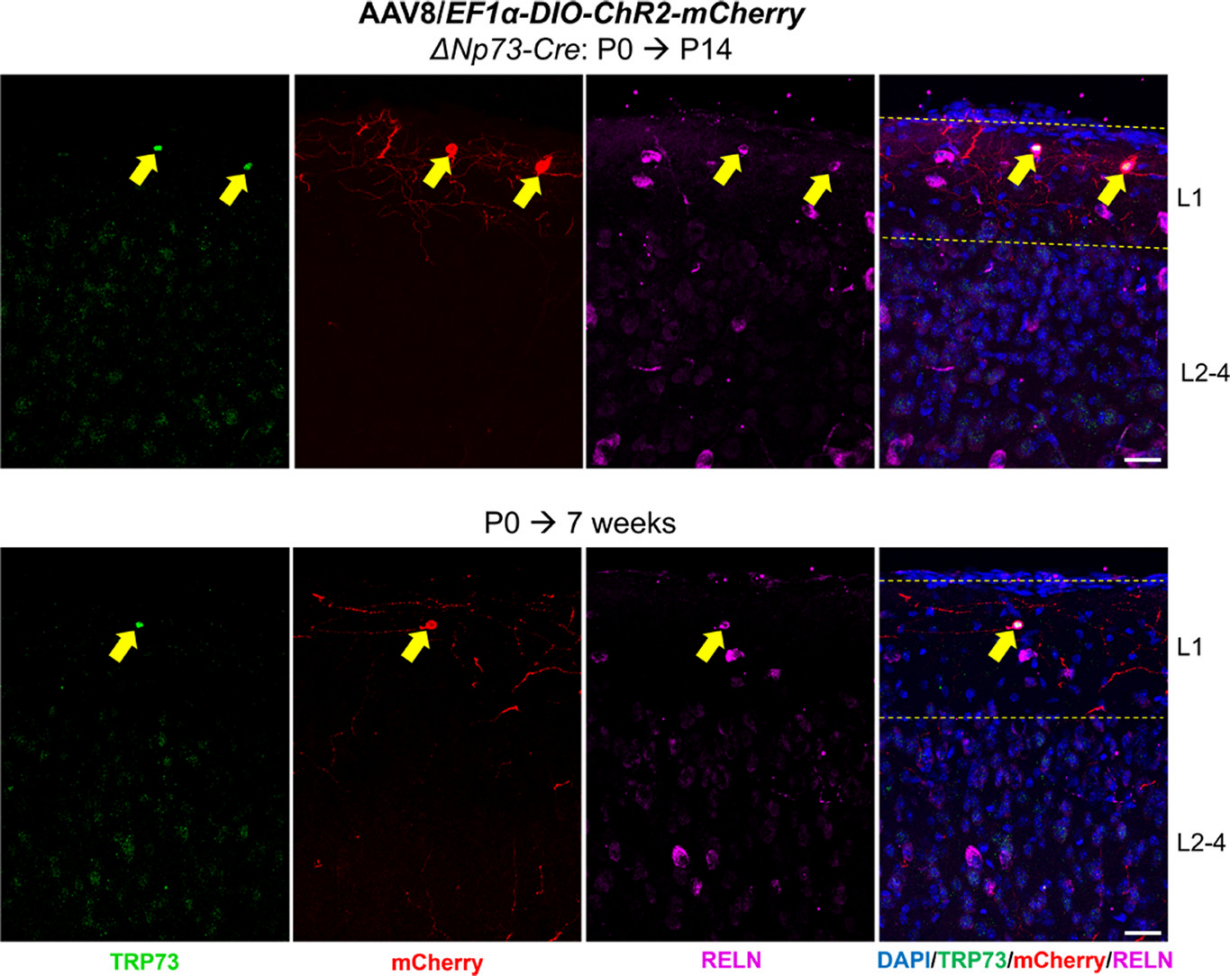
Effects of neonatal intraventricular adeno-associated virus injection on *ΔNp73*-lineage cells in the neocortex. Δ*Np73-Cre* pups injected at P0 with AAV8/*EF1a-DIO-ChR2-mCherry* were analyzed for mCherry expression at 14 d (P14, top) or 7 weeks (bottom) post injection. Coimmunostaining of TRP73 and RELN identifies neocortical Cajal–Retzius cells that express mCherry (yellow arrows). Neocortical Cajal–Retzius cells are only found in layer 1 (L1). They are very sparse at P14 and almost completely disappear by 7 weeks of age. Scale bars, 30 μm.

**Figure 10.**
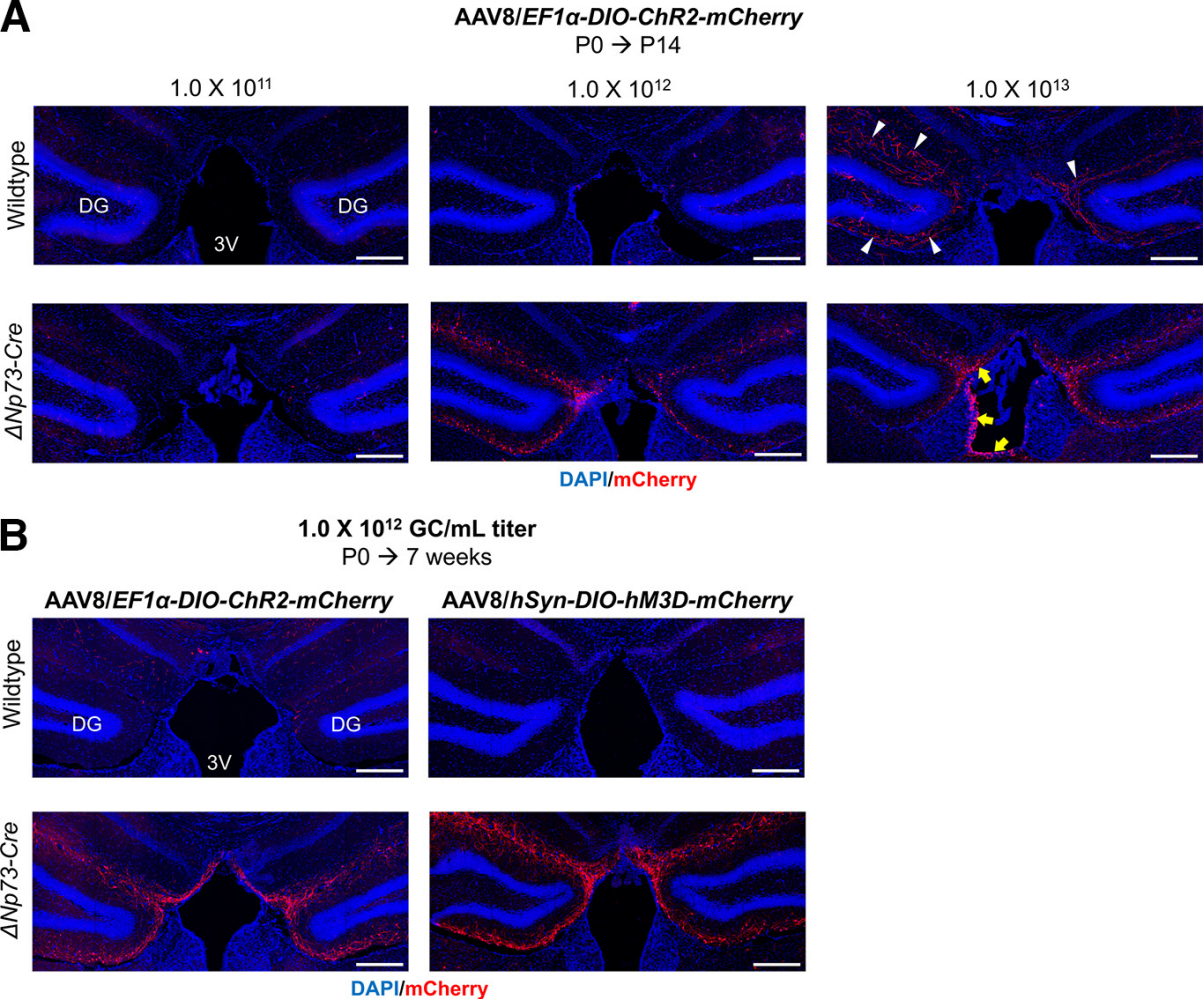
Effects of neonatal intraventricular adeno-associated virus injection on *ΔNp73*-lineage cells in the third ventricle and choroid plexus. ***A***, Wild-type or Δ*Np73-Cre* pups injected at P0 with AAV8/*EF1a-DIO-ChR2-mCherry* at different titers were analyzed for mCherry expression at P14. At 1.0 × 10^13^ viral titer, Cre-independent recombination was evident in the wild-type mice (white arrowheads). Additionally, at 1.0 × 10^13^ GC/ml viral titer, choroid plexus epithelial cells in the third ventricle (3V) of the Δ*Np73-Cre* mice were also transduced, evident from their mCherry expression (yellow arrows). Transduction of epithelial cells is not detectable at the 1.0 × 10^12^ AAV titer. Scale bars, 300 μm. ***B***, Wild-type or Δ*Np73-Cre* pups injected at P0 with AAV8/*EF1a-DIO-ChR2-mCherry* or AAV8/*hSyn-DIO-hM3D-mCherry* at 1.0 × 10^12^ GC/ml viral titer were analyzed for mCherry expression at 7 weeks postinjection. Reporter expression is absent from ependymal and choroid plexus cells.

Our study focuses on the Δ*Np73-Cre* line, because of its popularity in CR cell research (Tissir et al., 2009; Ledonne et al., 2016; Riva et al., 2019; Anstötz et al., 2022; Genescu et al., 2022) and our data showing its high specificity compared with the *Wnt3a-Cre* line. However, the homozygous Δ*Np73-Cre* mouse model results in knockout of *ΔNp73* and ablation of *ΔNp73*-lineage CR cells in the brain (Tissir et al., 2009). In our study, we used only the hemizygous Δ*Np73-Cre* mice, which do not demonstrate CR cell loss (Tissir et al., 2009) and present with similar CR cell densities in the hippocampus compared with wild-type mice. Moreover, while our study focuses only on the postnatal and adult hippocampus, we recognize that CR cells in the neocortex are also derived from the Δ*Np73* lineage (Ledonne et al., 2016; Riva et al., 2019; Genescu et al., 2022); therefore, our strategy will also transduce neocortical CRs. While very few neocortical CRs are present in the adult brain (Fig. 9), this is an important consideration when designing experiments, especially if the contribution of the few remaining neocortical CRs may lead to misinterpretation of results. Furthermore, choroid plexus and ependymal cells also belong to the Δ*Np73* lineage (Tissir et al., 2009; Marshall et al., 2016). In our hands, while the 1.0 × 10^13^ GC/ml viral titer did result in reporter expression within these cells, the lower 1.0 × 10^12^ GC/ml titer did not (Fig. 10), suggesting that careful titration of the AAV vector can reduce and even eliminate Cre-mediated recombination events in these cells.

Toward the goal of genetic manipulation of postnatal and adult CR cells, we adapted a previously developed neonatal intracerebroventricular injection method to introduce AAV vectors into the brain. Alternatively, AAVs can be introduced via stereotaxic injections. In a recent study, stereotaxic neonatal AAV injection was used to selectively ablate postnatal hippocampal CR cells in the *Pde1c-Cre* mice (Glærum et al., 2022). Compared with our fast and easy freehand neonatal injection method, stereotaxic neonatal injection is more labor intensive and time consuming, resulting in processing fewer animals per day, thereby increasing day-to-day variability. Stereotaxic neonatal injection also requires specialized surgery and injection equipment, and thus more technical training. While neonatal intracerebroventricular injection offers brain-wide transduction of the viral vector, the transduction pattern from stereotaxic injection is more localized, limiting to areas adjacent to the injection site (Kim et al., 2013). As CR cells are broadly distributed throughout the hippocampus, stereotaxic AAV injection may only transduce CR cells in a specific subregion of the hippocampus. However, such localized delivery may increase the local viral titer and allow for better control of the effective viral titer, which may explain the slightly higher transduction efficiency (∼70%) in the Glærum et al. (2022) study compared with our results (∼50%). However, this difference could also be because of the use of different Cre driver lines (*Pde1c-Cre* vs Δ*Np73-Cre*), which may have slightly different Cre expression levels and hence recombination efficiency in CR cells. Future studies applying our approach to the *Pde1c-Cre* mice or performing stereotaxic neonatal brain injections into the Δ*Np73-Cre* mice will provide a better comparison between the efficiencies of the two techniques and the two Cre-driver lines. In addition to neonatal AAV injection into Cre-driver mice, it may also be possible to genetically manipulate CR cells in the adult brain using the inducible Cre/ERT2 system, whereby Cre is activated after tamoxifen treatment (Metzger et al., 1995; Schwenk et al., 1998). The *Fzd10-Cre/ERT2* line shows inducible Cre-mediated recombination in CR cells during embryonic development (Metzger et al., 1995; Schwenk et al., 1998). However, its specificity for CR cells in the postnatal and adult brain has not been characterized, and therefore, its utility remains to be tested.

A limitation of our approach is that we only achieve Cre-mediated recombination in ∼50% of postnatal CR cells. This may limit the usefulness of our approach in conditional gene knock-out studies, both because of its relatively low efficiency and because the volume of the region transduced by the AAV may vary. Whether a ∼50% recombination rate is sufficient for gene overexpression, lineage tracing, circuit manipulation, and behavioral analysis should be evaluated on a case-by-case basis. In our hands, increasing the viral titer by 10-fold significantly increased viral transduction efficiency but led to increased animal morbidity and spontaneous recombination of the viral construct in DG granule neurons. Further optimization may be needed by using different Cre-driver lines and/or other AAV serotypes. Future studies may adapt our strategy to study the role of CR cells in the adult hippocampus via optogenetic and chemogenetic approaches. Previous studies have only stimulated CR cells optogenetically in *ex vivo* tissue (Quattrocolo and Maccaferri, 2014; Riva et al., 2019; Anstötz et al., 2022), or chemogenetically *in vivo* at early postnatal age from P1–P3 (Genescu et al., 2022). As a result, we know very little about the functions of CR cells in the adult brain circuit and animal behavior. Of note, we found that few CR cells in the adult hippocampus express c-Fos under basal condition, suggesting that they are inactive under the specific conditions of our experiment. Because of this low baseline activity, activating even a small number of CR cells using our approach may impact the hippocampal circuit and animal behavior. While optogenetic and chemogenetic manipulation of adult CR cells followed by behavioral studies is beyond the scope of our study, our framework offers a starting point for others to recognize the persistence of CR cells in the adult hippocampus and begin to address their functions.

## Synthesis

Reviewing Editor: Eran Perlson, Tel Aviv University Decisions are customarily a result of the Reviewing Editor and the peer reviewers coming together and discussing their recommendations until a consensus is reached. When revisions are invited, a fact-based synthesis statement explaining their decision and outlining what is needed to prepare a revision will be listed below. The following reviewer(s) agreed to reveal their identity: Alessandra Pierani.

The manuscript describes a new, simple, method for studying Cajal-Retzius cells. This will be useful for the field. Although the manuscript has improved, and the authors quantified nicely the experiments as requested, there are still some important points that need to be addressed by the authors

1) Throughout the manuscript, the authors tend to broadly generalize genetic crosses as nonspecific. This is not correct as the off-target recombination driven by the DNp73-Cre seem to occur only at later postnatal ages and quite inconsistently (not observed in every mouse). This should be explicitly clarified and all the sentences making broad generalization should be edited to indicate the complexity of the situation. For example, “(215-216) Neither Wnt3a-Cre nor DNp73-Cre lines are specific to Cajal-Retzius cells in the postnatal hippocampus” and “(274-276) our genetic crosses indicate that, while both Wnt3a-Cre and DNp73-Cre lines induce robust reporter expression in CR cells, they also lead to additional recombination in postnatal DG granule neuron” and “Cre-mediated recombination in dentate gyrus neurons driven by the DNp73-Cre allele is present in multiple reporter lines” directly contradict what stated by the authors “(236-237) At P7, tdTomato reporter expression was seen exclusively in CR cells in the DG”. Please correct all the sentences in the text making this generalization.

2) Illustrations. Instead of using different styles (For example, Figures 2B and 4D: violin plot for tdTomato+ TRP73+ CR cells vs plunger plots for tdTomato+ granule neurons), quantifications should be consistently illustrated by the same plot type and with the same scale limits to allow direct visual comparisons. Also, it would be helpful to add a graph directly quantifying the ratios of the numbers of tdTomato+ CR cells over tdTomato+ granule neurons (for each individual mouse), in order to directly show the degree of specificity. The authors already have these data, so adding this plot should be easy. As similar argument of for consistency of the plot type used can be made for the “variation between hemispheres” plots that in some figures use violin graphs and in others plunger plots.

3) I disagree with the authors’ suggestion that “(272) the DNp73-Cre allele may drive recombination in mature granule neurons stochastically”. I think that the data of the manuscript strongly argue against this possibility. Randomly occurring events in numerically large populations (such as hippocampal granule cells) would produce very similar averages. Statistically, the average number produced by many repeated dice throws is very consistent compared to just a few tosses. Here, the authors show a large variability of off-target recombination both between individual mice and even between left-right hemispheres of the same animals. It is more likely that off-target recombination is driven by individual differences in mice (behavior? physical activity? genetics?) rather than a generalized random recombination. Could the authors please discuss this point explicitly?

4) Cre-independent recombination and appearance of mCherry in DG granule neurons in mice injected with high viral titer. The authors suggest that this may be due to the “(371-372) Cre-independent spontaneous recombination of the viral vector”. However, the authors also state that they performed control experiments in wildtype mice and that “Cre-independent recombination rarely occurred and was limited to <2% of CR cells in the wildtype mice” injected with the virus (lines 376-380). If no Cre-independent recombination was observed in DG granule cells in wildtype mice, how can Cre-independent recombination occur DG granule cells of DN[73-Cre animals? This interpretation seems contradictory.

A simpler explanation that should be discussed is that the main difference between genetic crosses and viral injections is that viral injections trigger a much lower number of recombinations (indicated by the fact that only a relatively small percentage 40-45% of CR cells expressed mCherry) and hence will also result in a lower number of off-target effects. The authors clearly show that when the viral titer is increased, more recombinations are observed and hence more off-target effects are produced.

For this reason, the sentence “(398) This demonstrates that our approach of neonatal ICV AAV injection into DNp73-Cre pups effectively limits recombination events in granule neurons in adult mice” appears rather misleading, as the viral strategy limits recombinations BOTH in CR cells and granule neurons, so the results observed may not necessarily due to a higher specificity of the system, but to a general decrease of recombination events. Please clarify.

5) “(453) This suggests that adult hippocampal CR cells are normally inactive”, and “(558) suggesting that they are normally inactive”. These sentences seem too broad as cFos expression depends on the conditions of the animal and type of stimulus/activity received before the staining. “Normal” conditions include a large variety of stimuli. I would suggest clarifying that CR cells were inactive at the specific conditions of the experiment.

6) “(477) they are likely due to a “leaky” DNp73 promoter”. I find this explanation weak. If the DNp73 promoter was “leaky”, shouldn’t off-target recombination be similarly observed at all developmental ages and with little variability between animals?

7) “(495) it will be difficult to tease apart the contribution of the two cell types if both are manipulated simultaneously”. This sentence should be softened as it is not necessarily correct and depends on whether granule cells target the cells under study.

8) Another important limitation of this method that should be explicitly mentioned by the authors is that the relatively low efficiency (40-50%) of their system may limit its usefulness for conditional KO studies both because of its low efficiency and because its impact depends on the volume of the region affected by the viral injection (especially for in vivo studies).

9) Quantifications in Fig. 2B clearly show that DeltaNp73Cre expression in DG granule cells appears to robustly occur in 4-weeks old animals. Other ages do not show significant differences with respect to P7 (which has no expression in DG granule cells). Thus, all analyses and quantifications done after for other lines to show that the authors approach is more reliable should be tested at 4 weeks of age (or 5 weeks, although we do not know how this changes between 4 and 7 weeks). It is not clear how the authors can conclude that their approach using AAVs is better if at 7 weeks DG granule cells expression is not robustly detected. Moreover, the number of animals n=XXX is not mentioned in the figures legends for each experiments. In the Met p.10 lines 208-209 the authors state that « Animals without any tdTomato-expressing granule neurons were excluded from the granule neuron variation index calculation ». This is confusing and it remains unclear whether the authors properly assessed how many animals overall display DG granule cells expression at different ages and, thus, if the animals with « ectopic » DG expression represent a very small percentage.

10)The abstract is quite vague and the authors should more clearly state in it DeltaNp73 expression in DG/what stage (4 weeks), when AAVs injection can be specific for CRs and what they showed they can manipulate with this method/what age.

## Author Response

We thank the editor and reviewers for their positive responses. We have made all the requested changes. Our response to the reviewers’ comments and suggestions, as well as changes to the submitted manuscript are outlined in detail below (original referee comments are quoted in bold and italics, with our responses following in plain font; bolded text refers to the new version of the text and figures). Changes to the manuscript are highlighted in yellow in the revised manuscript.

Synthesis Statement for Author (Required):

The manuscript describes a new, simple, method for studying Cajal-Retzius cells. This will be useful for the field. Although the manuscript has improved, and the authors quantified nicely the experiments as requested, there are still some important points that need to be addressed by the authors

1) Throughout the manuscript, the authors tend to broadly generalize genetic crosses as nonspecific. This is not correct as the off-target recombination driven by the DNp73-Cre seem to occur only at later postnatal ages and quite inconsistently (not observed in every mouse). This should be explicitly clarified and all the sentences making broad generalization should be edited to indicate the complexity of the situation. For example, “(215-216) Neither Wnt3a-Cre nor DNp73-Cre lines are specific to Cajal-Retzius cells in the postnatal hippocampus” and “(274-276) our genetic crosses indicate that, while both Wnt3a-Cre and DNp73-Cre lines induce robust reporter expression in CR cells, they also lead to additional recombination in postnatal DG granule neuron” and “Cre-mediated recombination in dentate gyrus neurons driven by the DNp73-Cre allele is present in multiple reporter lines” directly contradict what stated by the authors “(236-237) At P7, tdTomato reporter expression was seen exclusively in CR cells in the DG”. Please correct all the sentences in the text making this generalization.

We have revised these statements as suggested so that they are less generalized and provide more specific context about the age of the animals (lines 214-215, 276-277, 279-280).

2) Illustrations. Instead of using different styles (For example, Figures 2B and 4D: violin plot for tdTomato+ TRP73+ CR cells vs plunger plots for tdTomato+ granule neurons), quantifications should be consistently illustrated by the same plot type and with the same scale limits to allow direct visual comparisons. Also, it would be helpful to add a graph directly quantifying the ratios of the numbers of tdTomato+ CR cells over tdTomato+ granule neurons (for each individual mouse), in order to directly show the degree of specificity. The authors already have these data, so adding this plot should be easy. As similar argument of for consistency of the plot type used can be made for the “variation between hemispheres” plots that in some figures use violin graphs and in others plunger plots.

We have made changes to the plot style so that all data are now presented in scatter (plunger) plots. Comparable datasets are presented using the same scale limits. We have also added quantification of CR cell-targeting specificity (the ratio of reporter-expressing CR cells over the sum of reporter-expressing CR cells and granule neurons) in Figs. 2B, 4D, and 6D as suggested and revised our results to reflect these new analyses (lines 260-261, 313-314, 398).

3) I disagree with the authors’ suggestion that “(272) the DNp73-Cre allele may drive recombination in mature granule neurons stochastically”. I think that the data of the manuscript strongly argue against this possibility. Randomly occurring events in numerically large populations (such as hippocampal granule cells) would produce very similar averages. Statistically, the average number produced by many repeated dice throws is very consistent compared to just a few tosses. Here, the authors show a large variability of off-target recombination both between individual mice and even between left-right hemispheres of the same animals. It is more likely that off-target recombination is driven by individual differences in mice (behavior? physical activity? genetics?) rather than a generalized random recombination. Could the authors please discuss this point explicitly?

We have revised the manuscript to discuss these possibilities explicitly (lines 272-273).

4) Cre-independent recombination and appearance of mCherry in DG granule neurons in mice injected with high viral titer. The authors suggest that this may be due to the “(371-372) Cre-independent spontaneous recombination of the viral vector”. However, the authors also state that they performed control experiments in wildtype mice and that “Cre-independent recombination rarely occurred and was limited to <2% of CR cells in the wildtype mice” injected with the virus (lines 376-380). If no Cre-independent recombination was observed in DG granule cells in wildtype mice, how can Cre-independent recombination occur DG granule cells of DN[73-Cre animals? This interpretation seems contradictory. A simpler explanation that should be discussed is that the main difference between genetic crosses and viral injections is that viral injections trigger a much lower number of recombinations (indicated by the fact that only a relatively small percentage 40-45% of CR cells expressed mCherry) and hence will also result in a lower number of off-target effects. The authors clearly show that when the viral titer is increased, more recombinations are observed and hence more off-target effects are produced.

For this reason, the sentence “(398) This demonstrates that our approach of neonatal ICV AAV injection into DNp73-Cre pups effectively limits recombination events in granule neurons in adult mice” appears rather misleading, as the viral strategy limits recombinations BOTH in CR cells and granule neurons, so the results observed may not necessarily due to a higher specificity of the system, but to a general decrease of recombination events. Please clarify.

We have revised our statement to clarify that both Cre-dependent and Cre-independent recombination may occur when viral titer is too high (lines 372-377). The reviewer raised an interesting possibility-lower AAV targeting efficiency and therefore less off-target effects. However, our new analysis of CR cell-targeting specificity in Fig. 6D argues against this possibility, as our AAV approach is more specific than genetic crosses.

5) “(453) This suggests that adult hippocampal CR cells are normally inactive”, and “(558) suggesting that they are normally inactive”. These sentences seem too broad as cFos expression depends on the conditions of the animal and type of stimulus/activity received before the staining. “Normal” conditions include a large variety of stimuli. I would suggest clarifying that CR cells were inactive at the specific conditions of the experiment.

We have revised these statements as suggested (lines 454-455 and line 562).

6) “(477) they are likely due to a “leaky” DNp73 promoter”. I find this explanation weak. If the DNp73 promoter was “leaky”, shouldn’t off-target recombination be similarly observed at all developmental ages and with little variability between animals?

We have removed the word “leaky” throughout the manuscript (lines 284, 287, 478-479).

7) “(495) it will be difficult to tease apart the contribution of the two cell types if both are manipulated simultaneously”. This sentence should be softened as it is not necessarily correct and depends on whether granule cells target the cells under study.

We have softened the sentence by replacing “it will be” with “it may be” (line 496).

8) Another important limitation of this method that should be explicitly mentioned by the authors is that the relatively low efficiency (40-50%) of their system may limit its usefulness for conditional KO studies both because of its low efficiency and because its impact depends on the volume of the region affected by the viral injection (especially for in vivo studies).

We now have discussed this explicitly in the revised manuscript (lines 548-550).

9) Quantifications in Fig. 2B clearly show that DeltaNp73Cre expression in DG granule cells appears to robustly occur in 4-weeks old animals. Other ages do not show significant differences with respect to P7 (which has no expression in DG granule cells). Thus, all analyses and quantifications done after for other lines to show that the authors approach is more reliable should be tested at 4 weeks of age (or 5 weeks, although we do not know how this changes between 4 and 7 weeks). It is not clear how the authors can conclude that their approach using AAVs is better if at 7 weeks DG granule cells expression is not robustly detected. Moreover, the number of animals n=XXX is not mentioned in the figures legends for each experiments. In the Met p.10 lines 208-209 the authors state that « Animals without any tdTomato-expressing granule neurons were excluded from the granule neuron variation index calculation ». This is confusing and it remains unclear whether the authors properly assessed how many animals overall display DG granule cells expression at different ages and, thus, if the animals with « ectopic » DG expression represent a very small percentage.

As suggested by the reviewer, we have included new analysis in the revised manuscript that show that CR cell-targeting specificity in P17, 4-week-, and 7-week-old animals is significantly lower than that in P7 (Fig. 2B, CR cell targeting specificity). We therefore use the same parameter to compare the results of 7-week-old animals from AAV injections and genetics crossing (Fig. 6D). These new analyses further strengthened the conclusion that our neonatal AAV injection approach is highly specific.

We agree that sample size information is important. To give readers immediate access to these numbers, we have chosen to add animal number (N = X) information in the figures, rather than in the legends.

We have clarified the number of animals excluded (three out of seven, lines 207-209). We had to exclude these animals because animals without any tdTomato-expressing granule neurons will give an invalid formula for the variation index calculation.

10)The abstract is quite vague and the authors should more clearly state in it DeltaNp73 expression in DG/what stage (4 weeks), when AAVs injection can be specific for CRs and what they showed they can manipulate with this method/what age.

We have revised the abstract to provide more details regarding the Cre line and the timing.
