# Supplementary figures and images for: A Versatile Strategy for Genetic Manipulation of Cajal–Retzius Cells in the Adult Mouse Hippocampus
Source: eNeuro. 2023 Oct 16;10(10):ENEURO.0054-23.2023. doi: 10.1523/ENEURO.0054-23.2023 (PMC10585607; doi:10.1523/ENEURO.0054-23.2023)

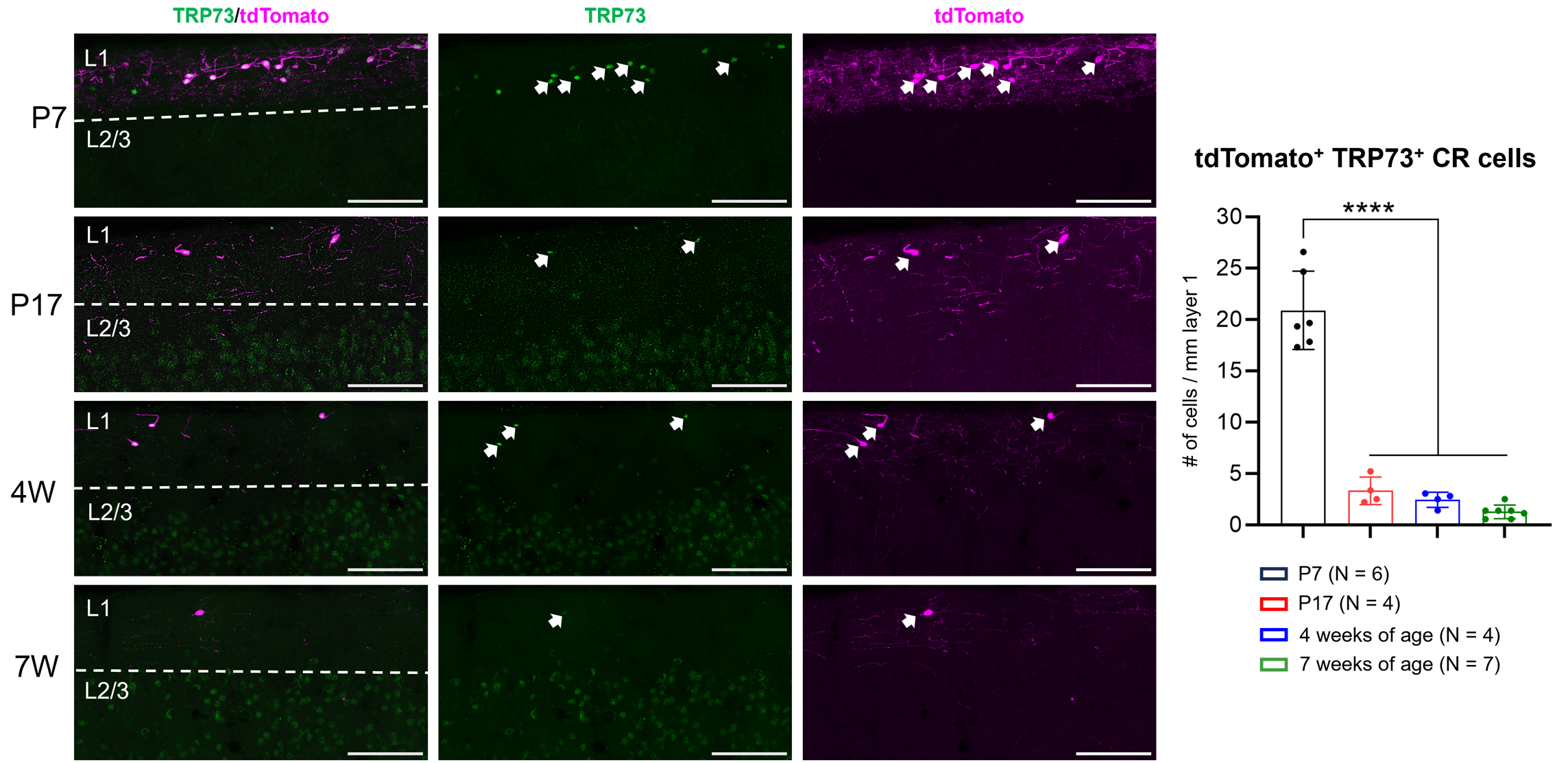

Supplement: Figure 2-1 — Reporter expression is restricted to Cajal–Retzius neurons in the neocortex of the ΔNp73-Cre; LSL-tdTomato mice. Left, Representative confocal images show coimmunostaining of the tdTomato reporter and the CR cell marker TRP73 in P7, P17, and 4-week-old (4W) and 7W mice. Arrows point to CR cells. Scale bars, 100 μm. L, Layer. Right, Quantification of cortical layer 1 CR cell density at different ages demonstrates the developmental program cell death of CRs. Data are presented as a scatter plot with all data points shown. Each data point is an individual animal, whereby three sections were measured for each animal. Statistical analyses were performed using nested one-way ANOVA with Tukey’s post hoc test. ****p < 0.0001. Download Figure 2-1, TIF file. [file enu-eN-MNT-0054-23-s02.tif]

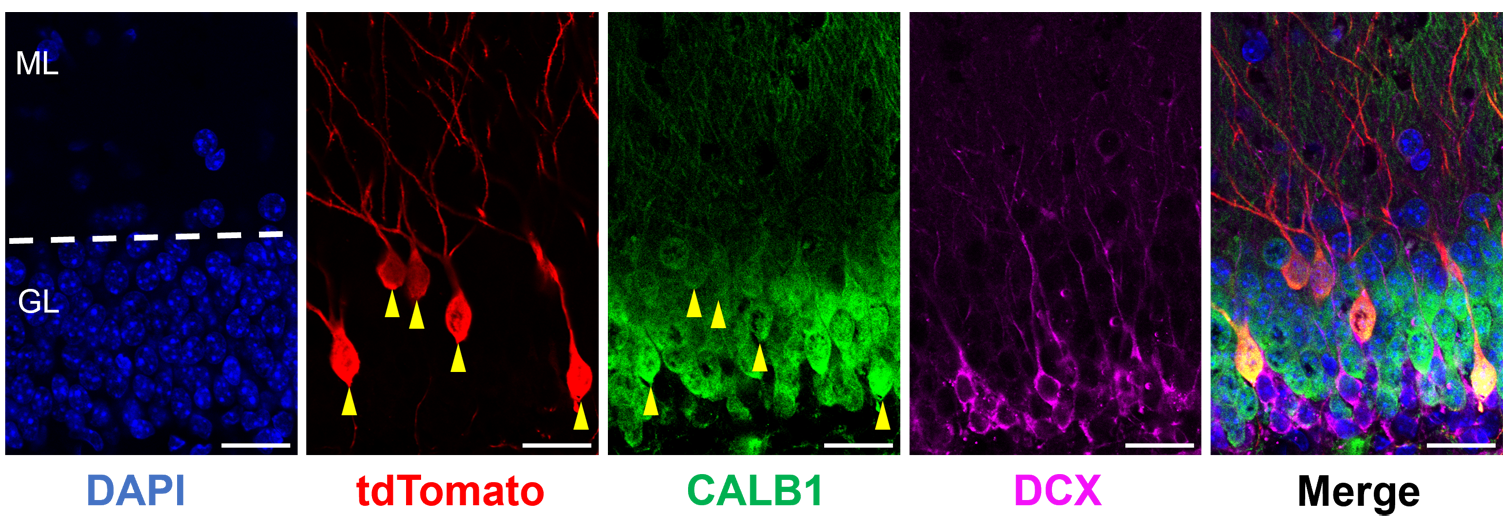

Supplement: Figure 2-2 — Recombination in mature granule neurons in the ΔNp73-Cre; LSL-tdTomato mice at 4 weeks of age. Coimmunostaining with the mature neuron marker CALB1 and the immature neuron marker DCX demonstrates that the tdTomato+ neurons in the granular layer (GL) are mature granule neurons (yellow arrowheads). Scale bars, 25 μm. ML, Molecular layer Download Figure 2-2, TIF file. [file enu-eN-MNT-0054-23-s03.tif]

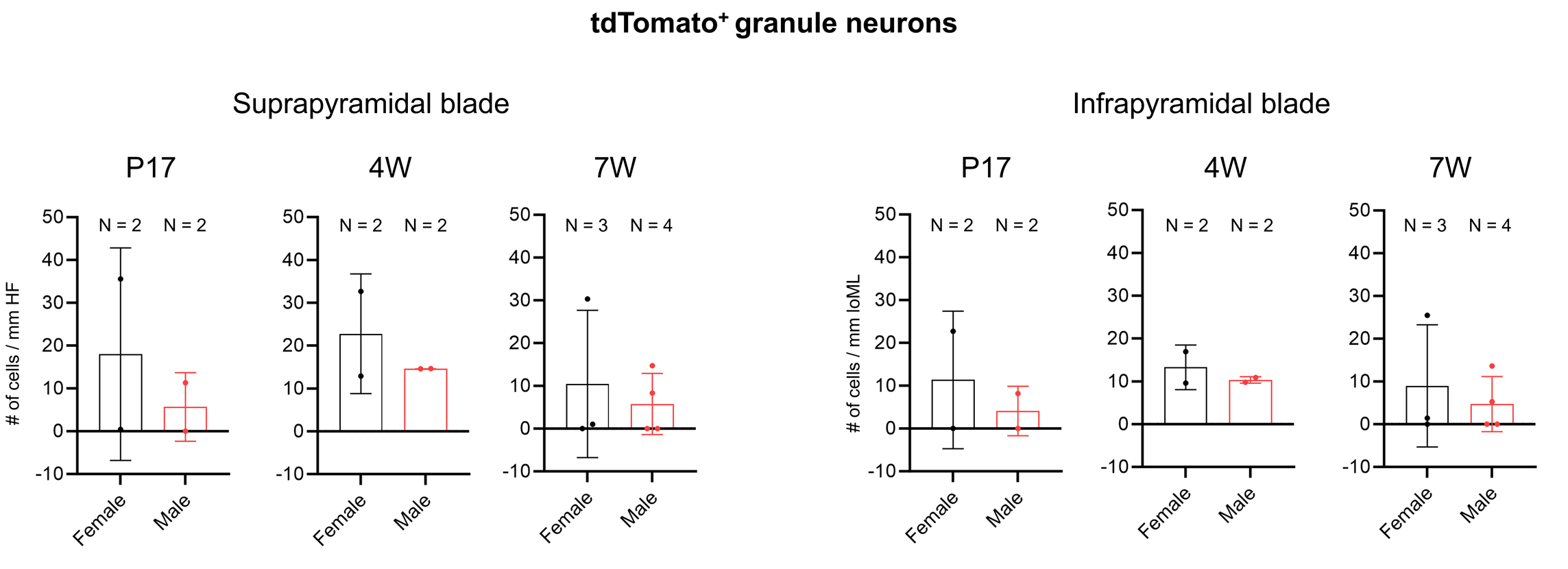

Supplement: Figure 2-3 — Analysis of potential sex effects on the variation of recombination in granule neurons in the ΔNp73-Cre; LSL-tdTomato mice. W, Weeks of age. Data are presented as scatter plots with all data points shown and error bars representing ±SD. Each data point is an individual animal, whereby three sections were measured for each animal. Statistical analyses were performed using nested t test. Download Figure 2-3, TIF file. [file enu-eN-MNT-0054-23-s04.tif]

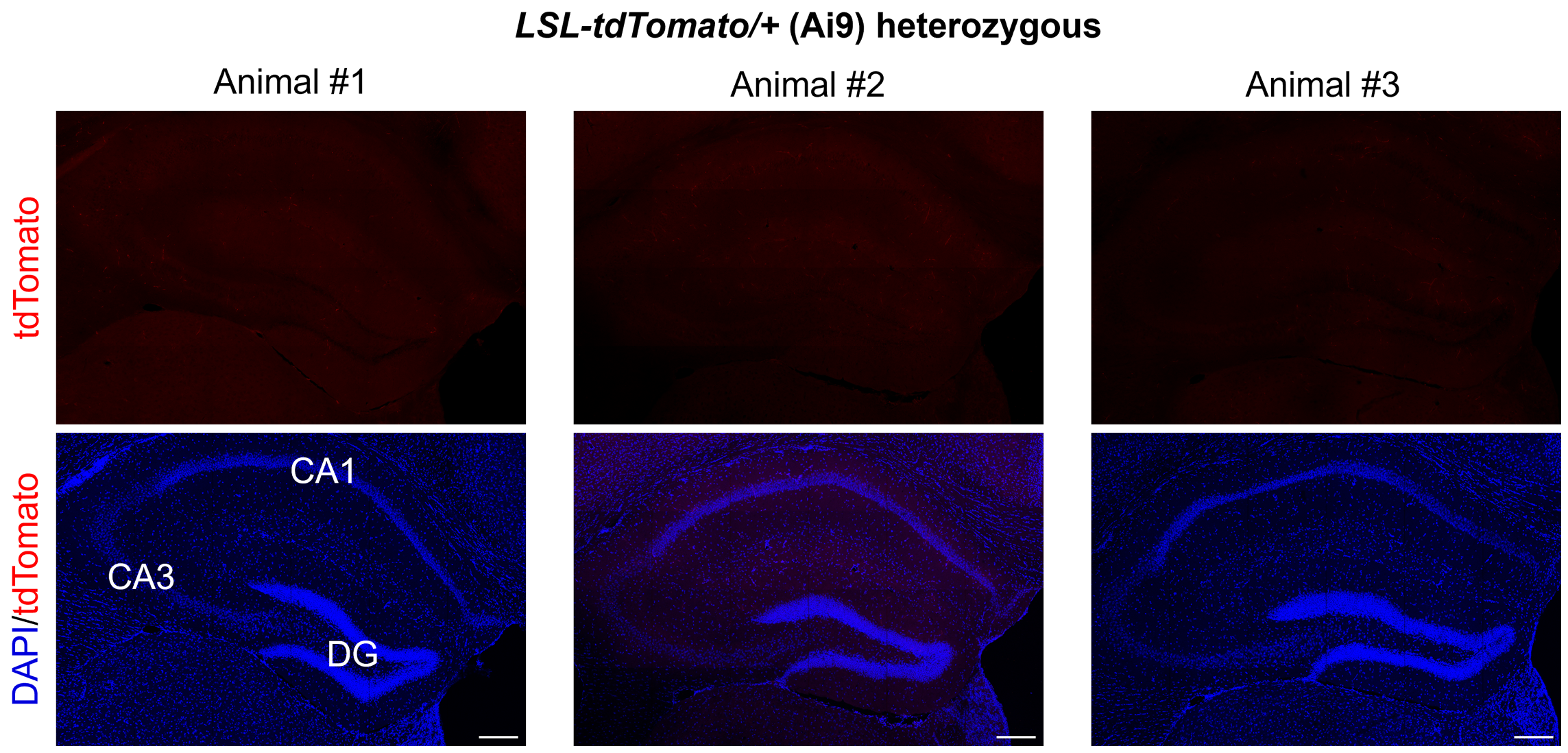

Supplement: Figure 4-1 — Absence of Cre-independent recombination events in the hippocampus of the LSL-tdTomato/+ heterozygous mice. Representative confocal images of immunostaining for tdTomato from three different animals are shown. A total of 4 animals (3 sections/animal) were examined. CA, Cornu ammonis. Scale bar, 200 μm. Download Figure 4-1, TIF file. [file enu-eN-MNT-0054-23-s05.tif]

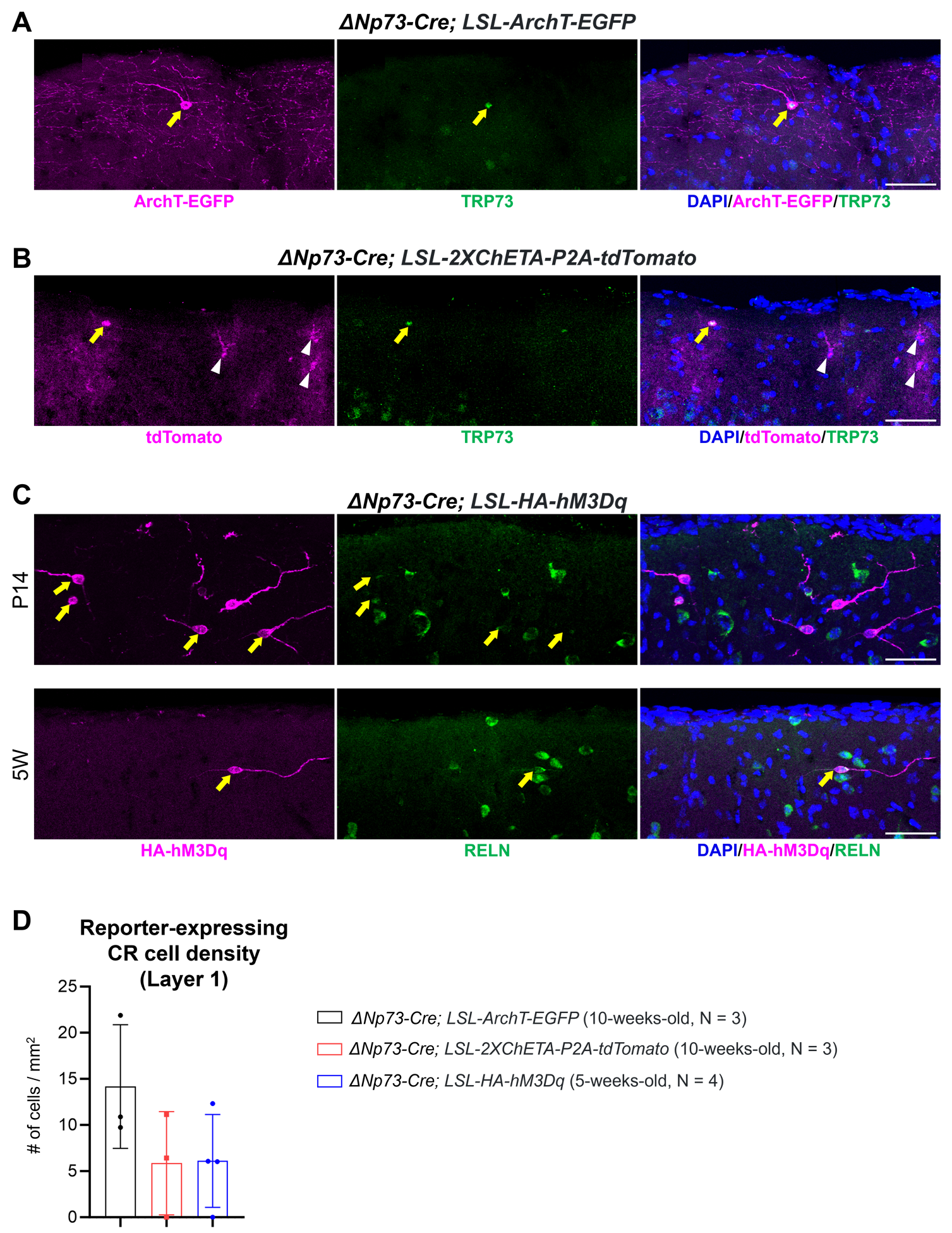

Supplement: Figure 4-2 — Reporter expression in neocortex layer 1 in different reporter lines. A, Representative confocal images show expression of the ArchT-EGFP reporter in a CR cell (yellow arrows) in layer 1 of a 10-week-old ΔNp73-Cre; LSL-ArchT-EGFP mouse. Reporter expression was present only in CR cells. Scale bar, 50 μm. B, Representative confocal images show the expression of the toTomato reporter in a CR cell in layer 1 of a 10-week-old ΔNp73-Cre; LSL-2XChETA-P2A-tdTomato mouse. Reporter expression was present not only in CR cells (yellow arrows) but also in non-CR cells in layer 1 (white yellowheads). Scale bar, 50 μm. C, Representative confocal images show expression of the HA-hM3Dq reporter in CR cells (yellow arrows) in layer 1 of a P14 (top) and a 5-week-old (bottom) ΔNp73-Cre; LSL-HA-hM3D mouse. Reporter expression was restricted to CR cells. Scale bar, 50 μm. D, Quantification of neocortical layer 1 reporter-expressing CR cell density of the three reporter lines. There was no significant difference between the groups. Data are presented as a scatter plot with all data points shown and error bars representing ±SD. Each data point is an individual animal, whereby three sections were measured for each animal. Statistical analysis was performed using nested one-way ANOVA with Tukey’s post hoc test. Download Figure 4-2, TIF file. [file enu-eN-MNT-0054-23-s06.tif]

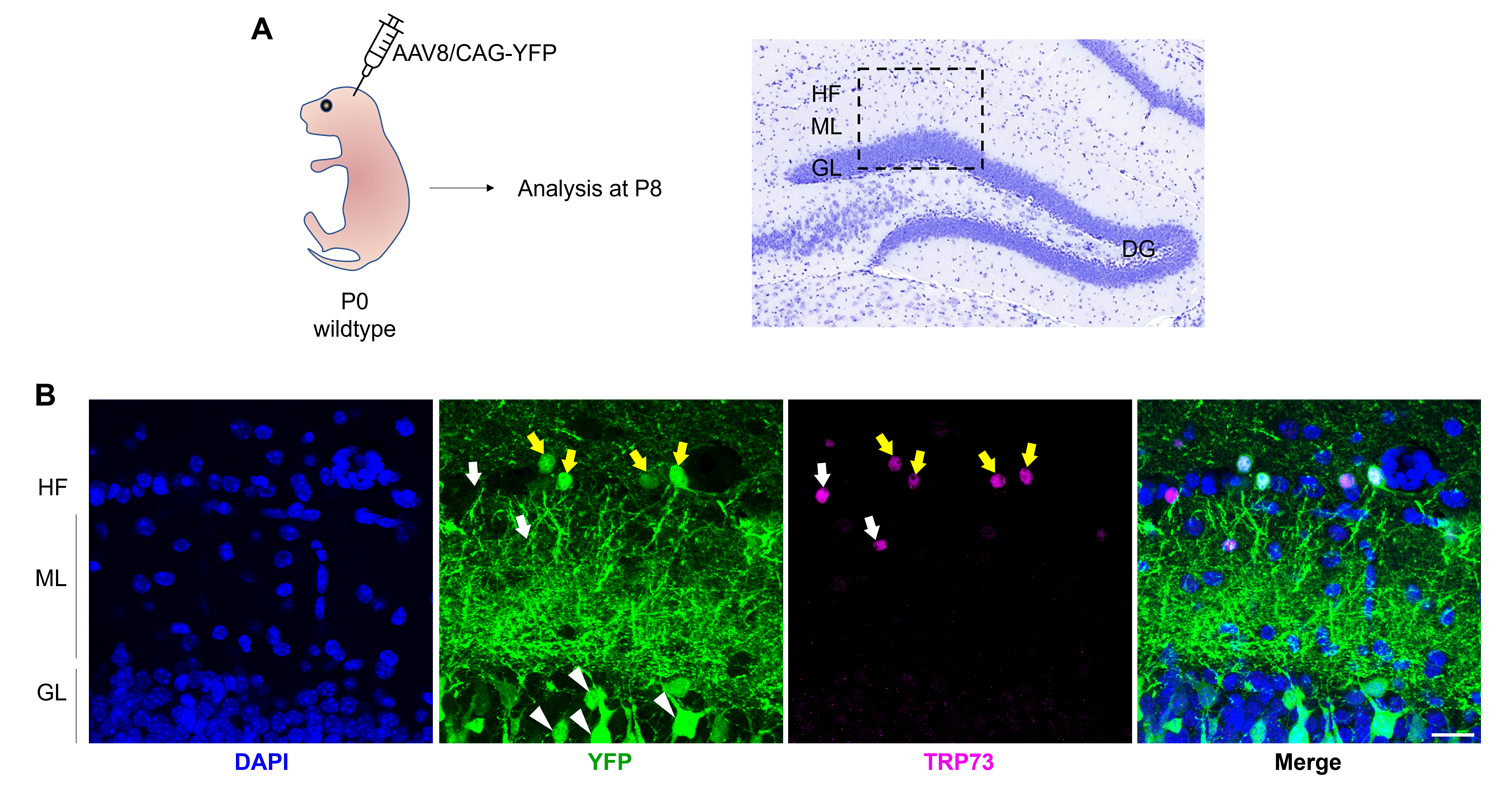

Supplement: Figure 5-1 — Adeno-associated virus serotype 8 efficiently transduces Cajal–Retzius cells when injected intraventricularly at postnatal day 0. A, Schematic shows the intraventricular injection and brain region of interest. Animals were injected at P0 with AAV8 carrying a YFP expression construct driven by the CAG promoter (CAG-YFP), and brain tissue was analyzed at P8. B, Many TRP73+ Cajal–Retzius cells express YFP (yellow arrows), while some do not (white arrows). Some granule neurons in the granular layer (GL, white arrowheads) also express YFP. HF, Hippocampal fissure; ML, molecular layer; GL, granular layer. Scale bar, 20 μm. Download Figure 5-1, TIF file. [file enu-eN-MNT-0054-23-s07.tif]

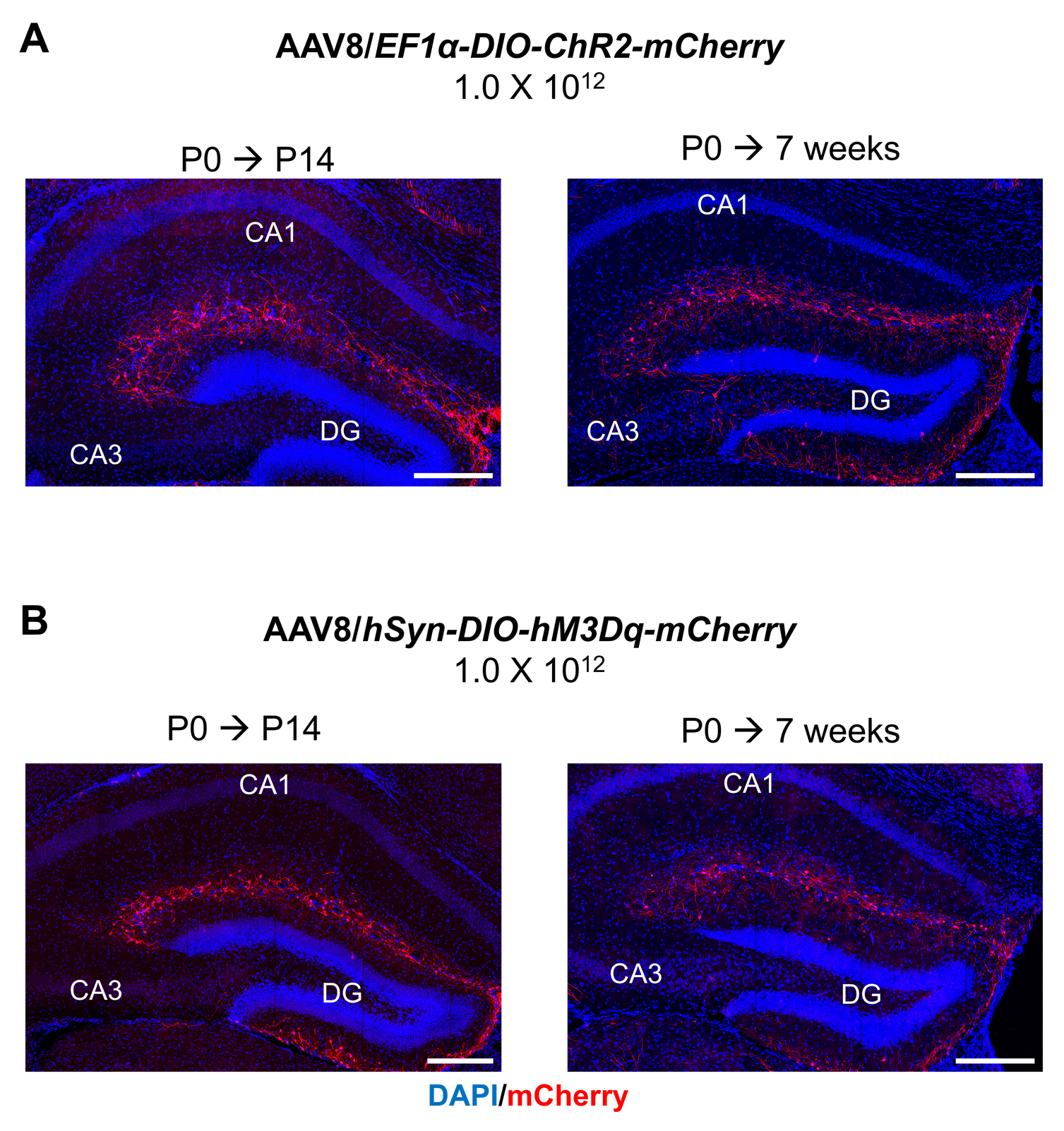

Supplement: Figure 5-2 — Neonatal intraventricular injection of Cre-dependent adeno-associated virus confers specificity in Cajal–Retzius cells. A, B, Neonatal (P0) ΔNp73-Cre pups were injected with 1.0 × 10^12^ GC/mL AAV8/EF1α-DIO-ChR2-mCherry (A) or AAV8/hSyn-DIO-hM3D-mCherry (B), and brain tissue analyzed at P14 or 7 weeks of age. CA, Cornu Ammonis. Scale bar, 300 μm. Download Figure 5-2, TIF file. [file enu-eN-MNT-0054-23-s08.tif]

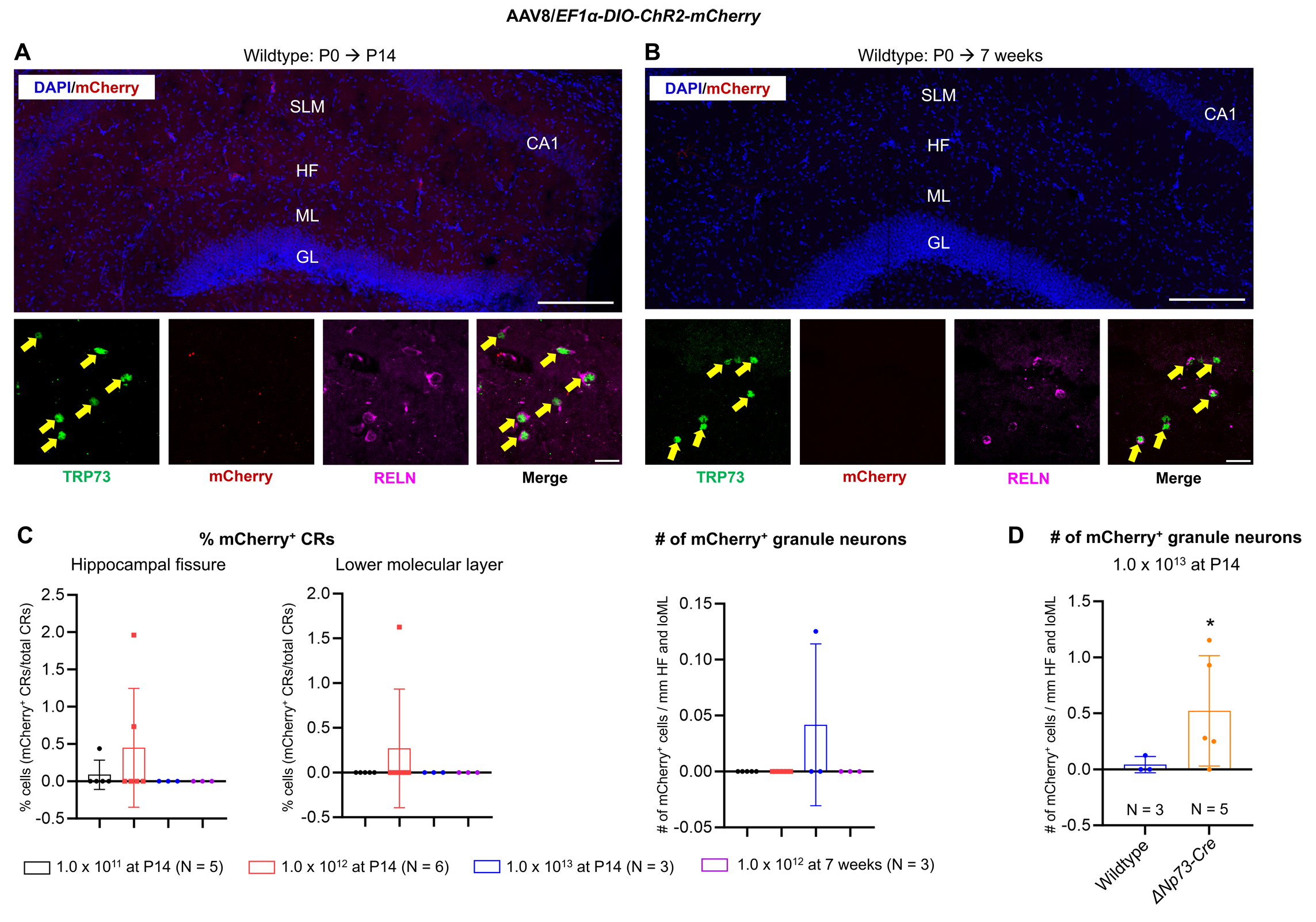

Supplement: Figure 5-3 — Absence of Cre-mediated recombination in Cajal–Retzius cells in wild-type mice neonatally injected with AAV8/EF1a-DIO-ChR2-mCherry. A, B, Wild-type pups (littermates of ΔNp73-Cre mice) injected at P0 with AAV8/EF1a-DIO-ChR2-mCherry were analyzed for mCherry expression at 14 days (P14; A) or 7 weeks (B) postinjection. Coimmunostaining of TRP73 and RELN identifies CR cells (yellow arrows), which do not express mCherry. Scale bars: top, 200 μm; bottom, 20 μm. SLM, Stratum lacunosum-moleculare; HF, hippocampal fissure; ML, molecular layer; GL, granular layer; CA, cornu ammonis. C, Quantification of the proportion of mCherry+ CR cells and the density of mCherry+ granule neurons. D, Quantification of CR cell-targeting specificity in P14 wild-type and ΔNp73-Cre mice injected with AAV8/EF1a-DIO-ChR2-mCherry at P0. Data are presented as scatter plots with all data points shown and error bars representing ±SD, and statistical analyses were performed using nested a t test or one-way ANOVA with Tukey’s post hoc test. Each data point is an individual animal, whereby three sections were measured for each animal. *p < 0.05. Download Figure 5-3, TIF file. [file enu-eN-MNT-0054-23-s09.tif]

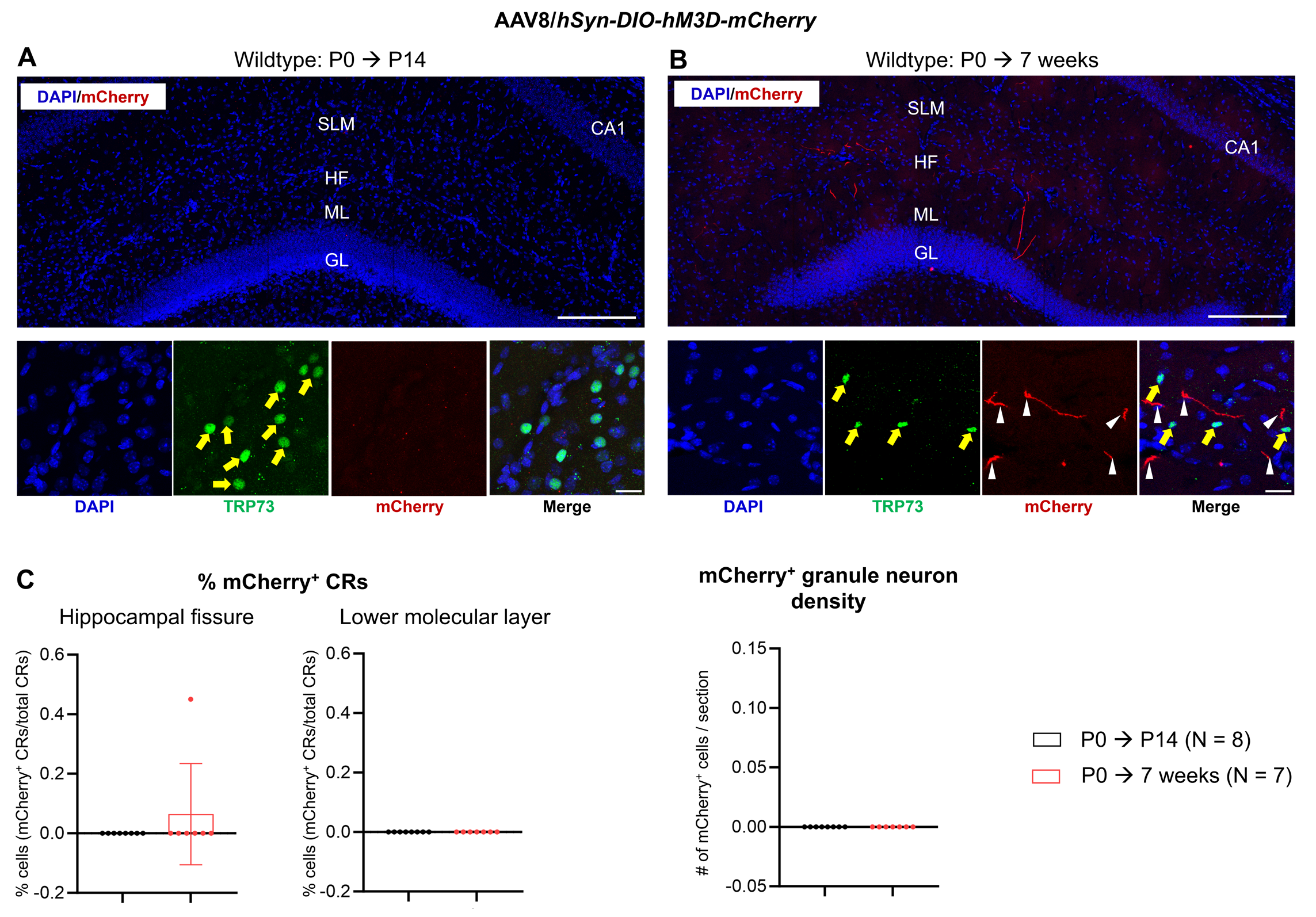

Supplement: Figure 7-1 — Absence of Cre-mediated recombination in Cajal–Retzius cells in wild-type mice neonatally injected with AAV8/hSyn-DIO-hM3D-mCherry. A, B, Wild-type pups (littermates of ΔNp73-Cre mice) injected at P0 with AAV8/hSyn-DIO-hM3D-mCherry were analyzed for mCherry expression at 14 days (P14; A) or 7 weeks (B) postinjection. Immunostaining of TRP73 identifies Cajal–Retzius cells (yellow arrows), which do not express mCherry. Some mCherry expression was observed in processes (white arrowheads) that do not belong to Cajal–Retzius cells. These were likely results of Cre-independent spontaneous recombination events of the viral vector during virus preparation, which is a well documented phenomenon for the double-floxed inverse orientation (DIO) system. SLM, Stratum lacunosum-moleculare; HF, hippocampal fissure; ML, molecular layer; GL, granular layer; CA, cornu ammonis. Scale bars: top, 200 μm; bottom, 20 μm. C, Quantification of the proportion of mCherry+ CR cells and the density of mCherry+ granule neurons. Data are presented as scatter plots with all data points shown and error bars representing ±SD, and statistical analyses were performed using nested one-way ANOVA with Tukey’s post hoc test. Each data point is an individual animal, whereby three sections were measured for each animal. Download Figure 7-1, TIF file. [file enu-eN-MNT-0054-23-s10.tif]

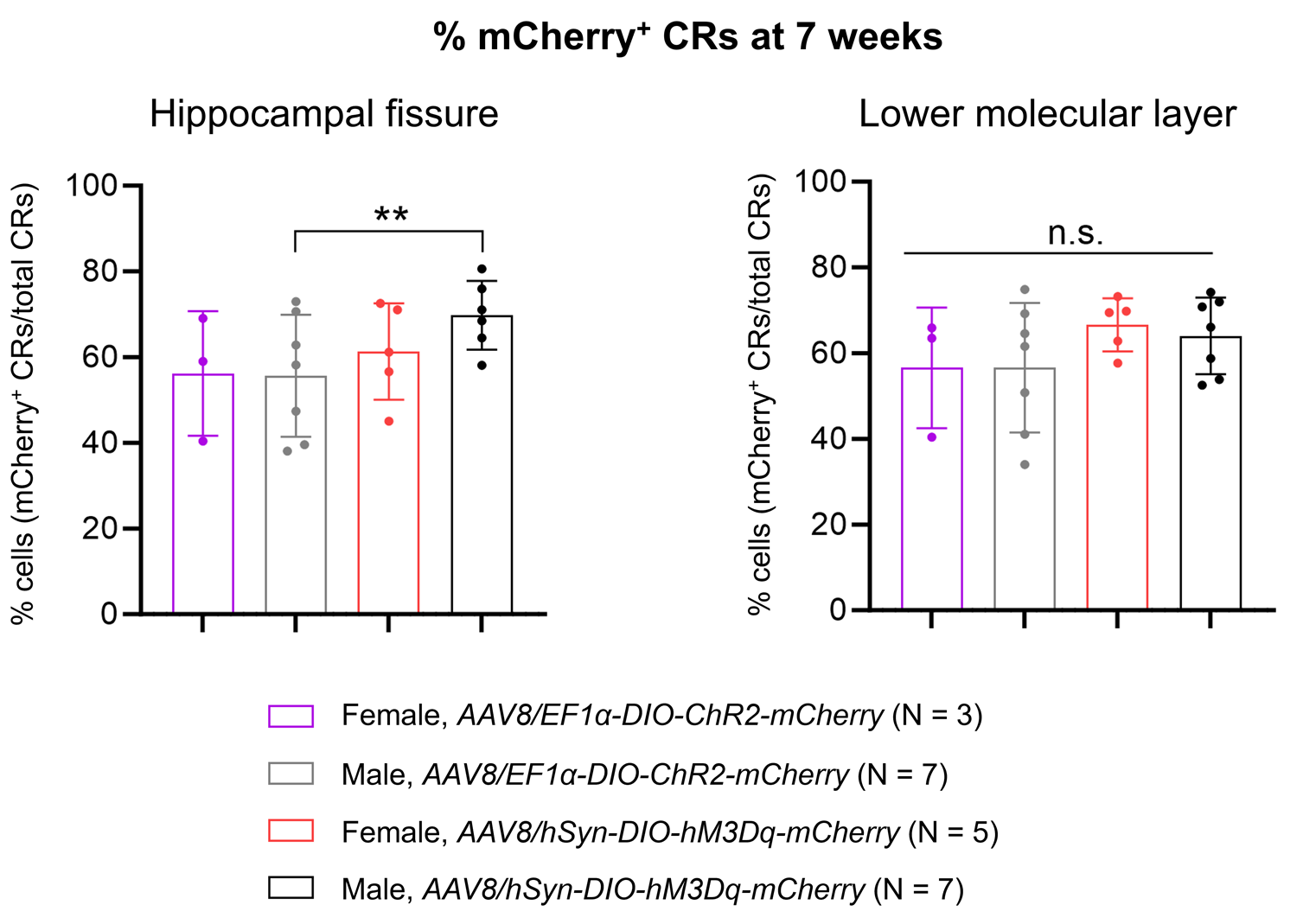

Supplement: Figure 7-2 — Analysis of potential sex effects on 7-week-old ΔNp73-Cre mice neonatally injected with AAV8/EF1α-DIO-ChR2-mCherry or AAV8/hSyn-DIO-hM3D-mCherry. In male mice, AAV8/hSyn-DIO-hM3D-mCherry transduced Cajal–Retzius cells along the hippocampal fissure more efficiently than AAV8/EF1α-DIO-ChR2-mCherry did. Data are presented as scatter plots with all data points shown. Each data point is an individual animal, whereby three sections were measured for each animal. Statistical analyses were performed using nested one-way ANOVA with Tukey’s post hoc test. **p < 0.01; n.s., not significant. Download Figure 7-2, TIF file. [file enu-eN-MNT-0054-23-s11.tif]

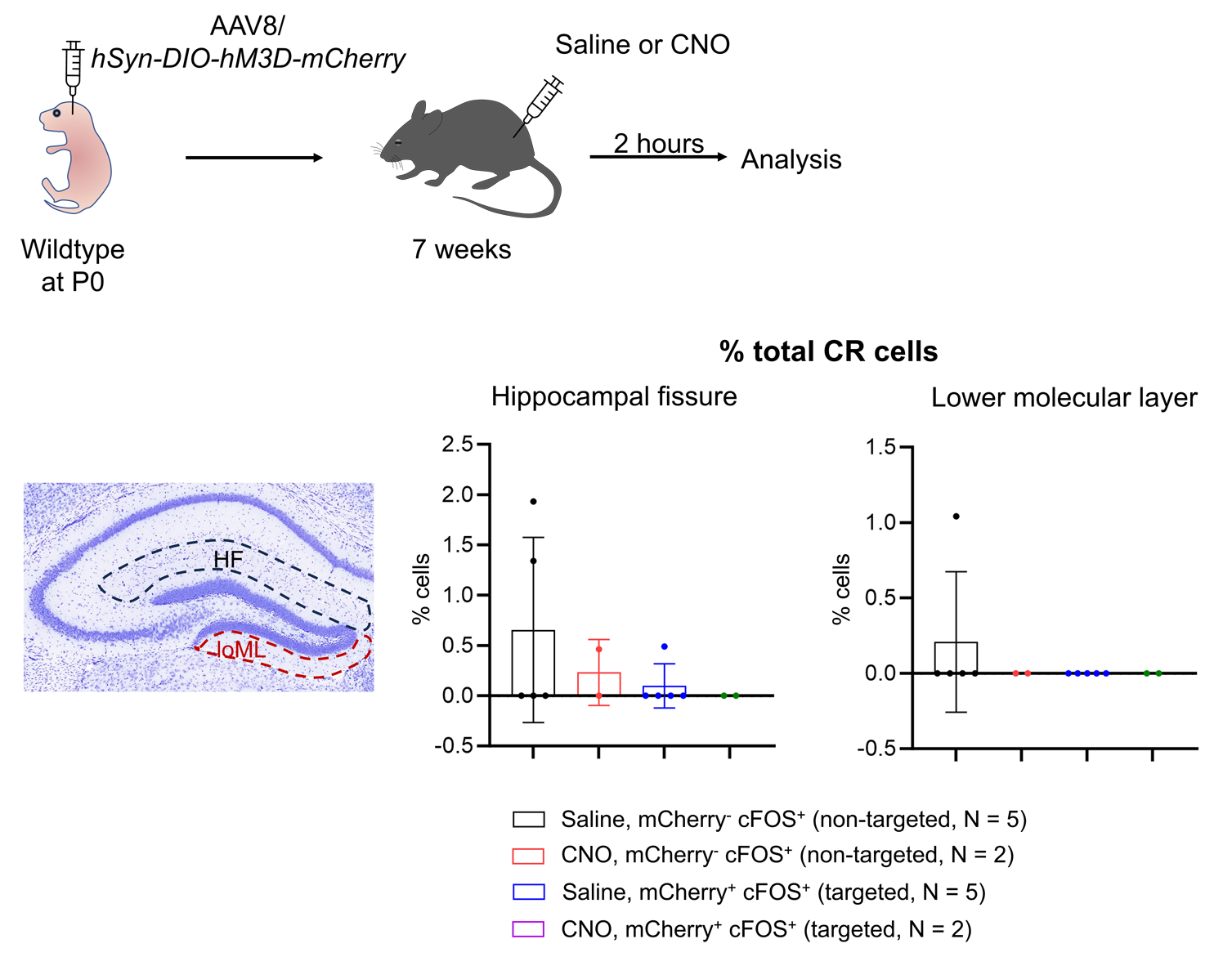

Supplement: Figure 8-1 — Cajal–Retzius cells in wild-type mice neonatally injected with the AAV8/hSyn-DIO-hM3D-mCherry are not activated by the chemogenetics approach. Top, Schematic of the experimental approach. Bottom, Quantification of the different groups of CR cells. Few CR cells were c-Fos+ in the wild-type mice regardless of treatment with saline or CNO. HF, Hippocampal fissure; loML, lower molecular layer (i.e., molecular layer of the infrapyramidal blade of the dentate gyrus). Data are presented as scatter plots with all data points shown and error bars representing ±SD. Each data point is an individual animal, whereby three sections were measured for each animal. Statistical analyses were performed using nested one-way ANOVA with Tukey’s post hoc test. Download Figure 8-1, TIF file. [file enu-eN-MNT-0054-23-s12.tif]
